# Integrating Multimodal Neuroimaging and Physical-Health Markers for Autism Spectrum Disorder in the ABCD Study

**DOI:** 10.31083/JIN48212

**Published:** 2026-02-26

**Authors:** Dorin Zeevi, Hector Acosta-Rodriguez, Pratheek Bobba, Alicia Stephan, Huang Lin, Ajay Malhotra, Seyedmehdi Payabvash

**Affiliations:** 1 Department of Radiology, Columbia University Irving Medical Center, New York Presbyterian Hospital, New York, NY 10032, USA; 2 Department of Radiology and Biomedical Imaging, Yale School of Medicine, New Haven, CT 06520, USA

**Keywords:** autism spectrum disorder, magnetic resonance imaging, diffusion magnetic resonance imaging, functional neuroimaging, sleep, machine learning, biomarkers

## Abstract

**Background:**

Autism Spectrum Disorder (ASD) is a complex neurodevelopmental condition characterized by diverse presentations, which complicates the identification of consistent biological markers. This study examined whether integrating multimodal neuroimaging and physical-health measures from a population-based cohort can improve ASD classification and reveal interpretable markers that reflect both clinical and community variation.

**Methods:**

Data were drawn from the Adolescent Brain Cognitive Development (ABCD) Study, a large community-based cohort of adolescents recruited from the general population. Participants with and without ASD were selected from this cohort, allowing contrasts that reflect natural variability across individuals. Structural, diffusion, and resting-state functional magnetic resonance imaging (MRI) data were integrated with physical-health markers, including sleep, growth, and early development. Propensity-score matching created demographically balanced groups, and multimodal machine learning models were evaluated through stratified cross-validation.

**Results:**

The multimodal integration of brain and physical-health markers outperformed single-modality models (area under the receiver operating characteristic curve [AUC-ROC] = 0.68, 95% confidence interval [CI]: 0.62–0.73; area under the precision-recall curve [AUC-PR] = 0.66, 95% CI: 0.60–0.73). Among physical-health markers, sleep function contributed most strongly to ASD classification, while neuroimaging predictors included cortical thickness in the right superior temporal gyrus and connectivity between the cingulo-opercular and default mode networks. These findings indicate that integrating modalities capturing both neural and physiological systems provides complementary information for identifying ASD-related differences within a population-based framework.

**Conclusions:**

This study provides a proof of concept that combining multimodal MRI and physical-health data within a large, demographically representative cohort can enhance ASD classification and yield biologically interpretable features. The population-based design situates these findings within a community context and offers a preliminary framework for integrating neural and physiological measures in future large-scale studies of neurodevelopmental diversity.

## 1. Introduction

Autism Spectrum Disorder (ASD) is a neurodevelopmental condition defined by persistent challenges in social communication and restricted, repetitive behaviors. Research demonstrates that ASD encompasses diverse presentations, with distinct underlying mechanisms influenced by genetic, epigenetic, and environmental factors [1,2]. Theconditionfrequentlypresentsalongsideintellectualdisabilities, language impairments, and comorbidities such as epilepsy, sleep disturbances, anxiety, and depression [3,4]. These complexities underscore the need to better understand ASD’s underlying neurobiological mechanisms. Diagnostic advances and increased public awareness have led to substantial changes in ASD prevalence rates. Data from the Centers for Disease Control and Prevention (CDC) indicate that ASD affected approximately 1 in 36 children (~2.8%) in 2020 in the United States, up from 1 in 150 (~0.7%) in 2000 [5]. Despite improved identification, the neurobiological underpinnings of ASD remain incompletely understood, with evidence implicating alterations in neural connectivity and synaptic function as key factors underlying social communication, behavioral differences, and sensory processing [6,7].

Neuroimaging research has advanced our understanding of ASD’s neural basis. Structural magnetic resonance imaging (s-MRI) has identified variations in cortical thickness, gray matter volume, and brain morphology, particularly in regions implicated in social cognition [8]. Diffusion MRI (d-MRI) studies have revealed atypical white matter integrity in tracts like the corpus callosum and arcuate fasciculus, which are critical for language and social processing [9,10]. Functional MRI (f-MRI) has demonstrated altered coordination within key brain networks during social, emotional, and language tasks [11,12]. However, ASD’s heterogeneity has complicated the identification of universal biomarkers. Large-scale population datasets, such as the Adolescent Brain Cognitive Development (ABCD) Study, offer a promising opportunity to integrate multimodal imaging with behavioral, genetic, and environmental data. These approaches may improve our understanding of ASD’s mechanisms, address its variability, and guide the development of targeted diagnostic and therapeutic strategies.

Our study leverages the population-based ABCD dataset to evaluate how multimodal markers contribute to ASD classification. We analyze structural, diffusion, and functional MRI (s-MRI, d-MRI, f-MRI) alongside physical-health indicators including sleep patterns, anthropometric measurements, and developmental history. Unlike traditional clinical cohorts, the ABCD dataset’s broad, representative sample enables a more generalizable investigation of ASD biomarkers. Through machine learning methods, we aim to identify the most significant biomarkers and assess the benefits of integrating multiple data types.

Our research objectives are threefold: (1) quantify the predictive value of different data modalities through a comprehensive ablation study, (2) apply propensity-score matching to mitigate confounding factors and dataset imbalances, and (3) identify and rank the most distinctive biomarkers across neuroimaging and physical-health measures. By leveraging the scale and diversity of the ABCD dataset, this study advances our understanding of ASD’s biological variability and underscores the importance of multimodal approaches in capturing ASD’s neurobiological and physiological complexity.

## 2. Materials and Methods

### 2.1 Dataset and Cohort Definition

#### 2.1.1 ABCD Study Overview

Our retrospective analysis focused on the study cohort from Release 5.1 of The ABCD Study, the largest longitudinal study in the United States spanning 21 research sites. The ABCD Study gathers broad range data on physical health, brain imaging, neurocognition, cultural and environmental factors, among other variables, from 11,868 children aged 9–10 at their baseline visit, recorded between September 2016 and January 2022 (https://abcdstudy.org/about/).

#### 2.1.2 ASD Case Ascertainment in ABCD

To determine eligibility for participation in the ABCD study, parents or caregivers underwent a screening interview where they provided general information about their children. As part of this initial screening, they were asked, “Has your child been diagnosed with Autism Spectrum Disorder?”. This parental report of ASD, previously utilized in multiple studies [13,14], has demonstrated consistency with known neurodevelopmental and psychopathology-related correlates of ASD at the population level [15]. In particular, prior work has shown that parent-reported ASD diagnosis in ABCD predicts downstream clinical outcomes consistent with established developmental risk trajectories. Of the 11,868 children in the ABCD dataset, 201 were reported to have ASD.

### 2.2 Neuroimaging Biomarkers

Children underwent baseline brain imaging and subsequent scans every other year, capturing both structural and functional aspects critical to adolescent development. The imaging protocol was divided into three main stages: (i) Pre-scan (outside scanner), where children were evaluated for MRI suitability and familiarized with the scanning process and associated tasks using simulators; (ii) During-scan (inside scanner), where children either participated in a single continuous session lasting 90–120 minutes or underwent two separate sessions with a break, totaling 100–120 minutes. This arrangement was determined by the operational flexibility of the imaging site or the scheduling needs of the families, allowing for the collection of both structural and functional images in a single visit; (iii) Post-scan (outside scanner), where children completed questionnaires and participated in memory tasks [16].

The imaging protocol included s-MRI with T1 and T2-weighted images, d-MRI featuring restriction spectrum imaging (RSI) and diffusion tensor imaging (DTI), and fMRI with four series in resting-state and two during task-based activities. Further details on the scans and biomarkers used in our study are discussed in the following sections. For a comprehensive review of the imaging protocols and procedures used in the ABCD study, please see references [16,17].

#### 2.2.1 Structural MRI (s-MRI)

Morphometric and macrostructural biomarkers of 34 cortical regions of interest (ROIs), as outlined in the Desikan-Killiany MRI atlas [18], were derived from high-resolution 1 × 1 × 1 mm T1-weighted scans. These biomarkers included cortical volume, thickness, surface areas, and sulcal depths at each brain hemisphere [17].

#### 2.2.2 Diffusion MRI (DTI and RSI)

Diffusion biomarkers for 23 primary white matter fiber tracts specified in AtlasTrack [19], and an additional 19 tracts not previously described, were derived from high angular resolution diffusion imaging (HARDI) with 1.7 × 1.7 × 1.7 mm scans, incorporating multiple b-values and fast integrated B0 distortion correction [17]. Two types of diffusion biomarkers were evaluated: (i) microstructural tissue properties, including fractional anisotropy and diffusivities (mean, longitudinal, and transverse), derived from full shell DTI; and (ii) intracellular (restricted) diffusion properties, which were modeled using distinct fiber orientation densities through RSI to assess multiple diffusion orientations within each voxel. DTI and RSI biomarkers were averaged for white matter tract ROIs identified by AtlasTrack and for 12 ROIs from automated subcortical segmentation conducted using FreeSurfer (version 5.3; Martinos Center for Biomedical Imaging, Massachusetts General Hospital, Boston, MA, USA).

#### 2.2.3 Resting-State f-MRI Connectivity

Functional connectivity biomarkers at resting-state for 12 distinct brain ROIs grouped into functional communities, as identified in the Gordon parcel atlas [20], were extracted during four 5-minute sessions using scans with a resolution of 2.4 × 2.4 × 2.4 mm and a Repetition Time (TR) of 800 milliseconds. Correlation values for each pair of ROIs were calculated, transformed into z-statistics via Fisher transformation, and averaged within and between networks to evaluate network correlation strength [17].

#### 2.2.4 Imaging Quality Assessment

As part of the imaging acquisition process, the ABCD Consortium conducted both visual and quantitative quality control analyses for each imaging modality to detect artifacts or abnormalities that could impair image readability. Quality control outcomes were summarized as a binary indication at the scan level to recommend whether it should be included [17]. Additionally, MRI scans were assessed for incidental findings based on neuroradiological reviews of the structural MRI images, categorizing them into one of five indications: 0 = Image artifacts prevent radiology read; 1 = No abnormal findings; 2 = Normal anatomical variant of no clinical significance; 3 = Consider clinical referral; 4 = Consider immediate clinical referral.

### 2.3 Physical-Health Markers

Physical-health information in the ABCD dataset was obtained from reports by parents or the children themselves [21]. We selected the following markers for inclusion: anthropometrics, developmental history, and sleep function, as detailed below.

#### 2.3.1 Anthropometrics

Anthropometric metrics were collected at ABCD baseline since obesity was shown to be an important factor in physical and mental health [21]. We used height (in), weight (lb), and waist circumference (in). When three measures were available, the two closest were averaged. Body mass index (BMI) was computed from height and weight.

#### 2.3.2 Developmental History

Information on each child’s developmental history was collected at baseline from parents using the questionnaire developed by the Adolescent Component of the National Comorbidity Survey [22,23]. This questionnaire covers events occurring during pregnancy and early childhood development. From this instrument, we selected three developmental markers previously linked to ASD: (i) maternal age at birth [24] (Question: “How old were you/biological mother when the child was born?”); (ii) premature birth (Question: “Was the child born prematurely?”); and (iii) extent of prematurity (Question: “About how many weeks premature was the child when they were born?”) [21].

#### 2.3.3 Sleep Function (SDSC)

Sleep function has been previously shown to be associated with ASD [25,26]. In the ABCD study, sleep function was evaluated using the Sleep Disturbance Scale for Children (SDSC), selected for its relevance to the age range of the participants. The scale includes 26 items, categorized into six sections: (1) disorders of initiating and maintaining sleep, (2) sleep breathing disorders, (3) arousal disorders or nightmares, (4) sleep-wake transition disorders, (5) excessive somnolence, and (6) sleep hyperhidrosis. The ABCD dataset provides a total score for each category and a composite score for the entire questionnaire, utilizing a total of 33 sleep function markers.

### 2.4 Inclusion/Exclusion Criteria

#### Participant Selection

**Fig. 1.**
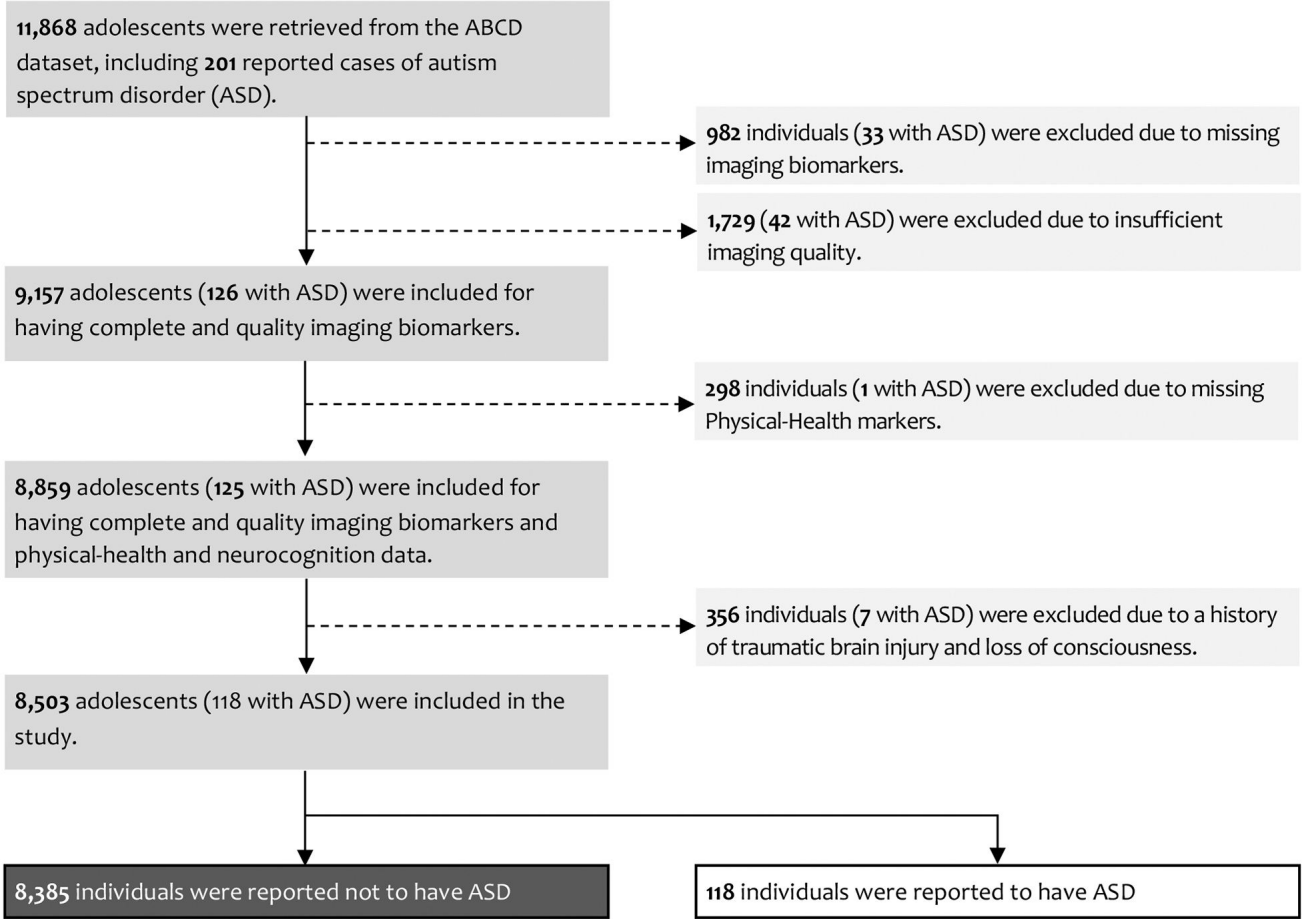
Cohort selection and exclusion criteria. Flowchart illustrating participant inclusion and exclusion from the Adolescent Brain Cognitive Development (ABCD) dataset. Of 11,868 adolescents (201 with autism spectrum disorder [ASD]), exclusions were applied for missing or low-quality imaging data, missing physical-health markers, and history of traumatic brain injury or loss of consciousness. The final analytic sample comprised 8503 participants (118 with ASD).

In this study, we selected subjects from the ABCD dataset who had complete physical-health and imaging biomarkers, during the initial ABCD baseline data collection. Subjects were excluded from the analysis if their imaging scans were not recommended for inclusion by the ABCD quality control, if image artifacts prevented radiological reading, or if they required immediate clinical referral following a radiology review. To control for confounding effects on brain structure and function, subjects with a history of traumatic brain injury or loss of consciousness as indicated by the Ohio State TBI Screen-Short ABCD data were excluded from the study. The cohort selection process, including exclusion criteria and participant numbers, is detailed in Fig. 1. Additionally, the Supplementary Material provides detailed information on the markers used, including their names, descriptions, and modalities as described in the ABCD data repository from which the data were extracted.

### 2.5 Marker Sets and Experimental Conditions

#### Uni- vs. Multimodal Definitions

We categorized markers into unimodal and multimodal groups to explore both the separate and combined contributions of imaging and physical-health markers in distinguishing ASD. For imaging biomarkers, we employed individual modalities including f-MRI, s-MRI, DTI, and RSI, as well as a comprehensive combination that integrates all these modalities. Similarly, for physical-health markers, we assessed the effects of anthropometrics, medical history, and SDSC markers individually and in combination. We also explored a cross-modality combination that includes both imaging and physical-health markers.

### 2.6 Modeling and Evaluation Pipeline

**Fig. 2.**
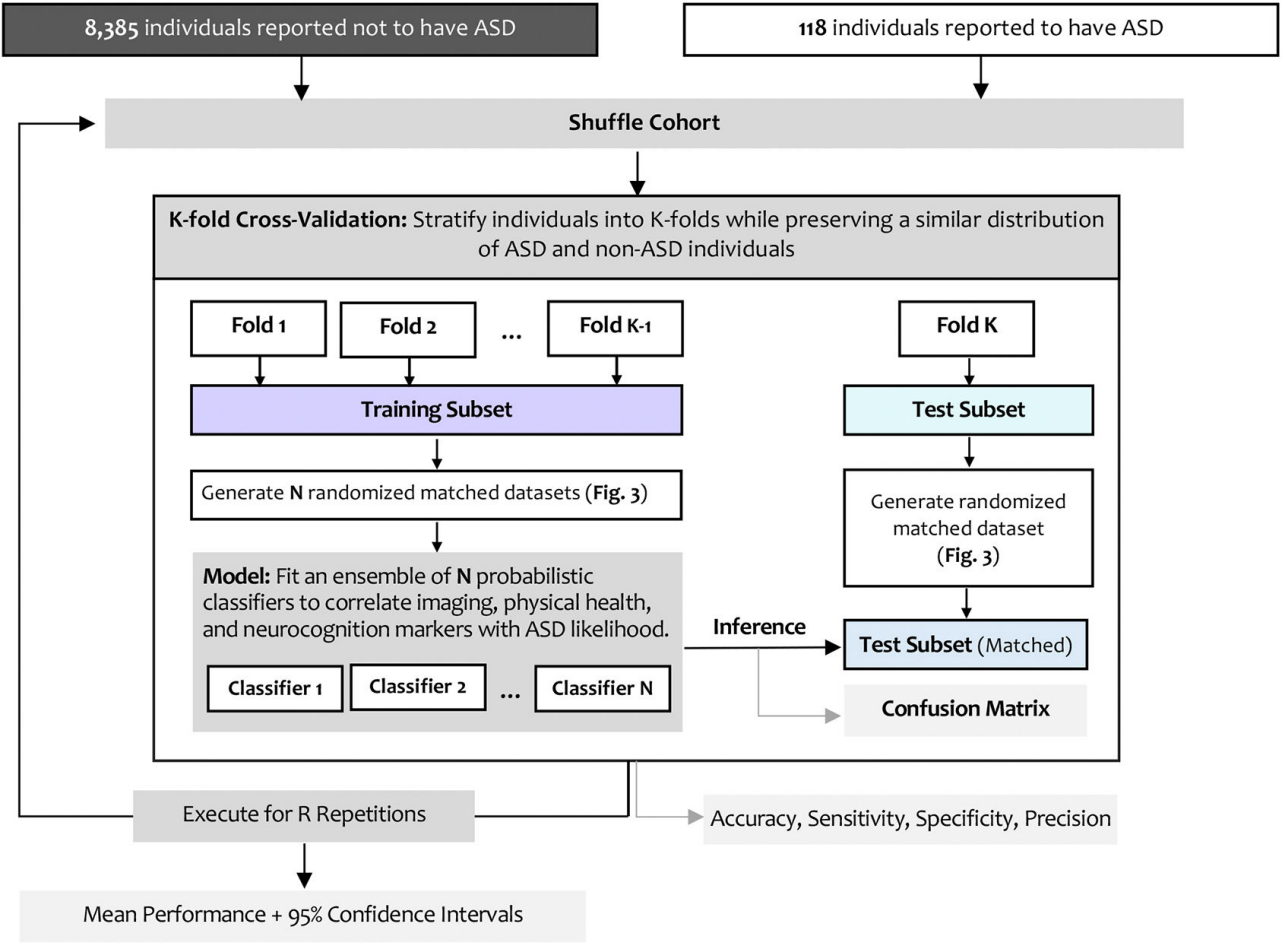
Modeling pipeline for ASD classification. Diagram summarizing the analytical workflow. Participants were divided into K-fold cross-validation subsets while preserving ASD/non-ASD ratios. Within each training fold, multiple randomized matched datasets were generated using propensity-score matching. Ensembles of probabilistic classifiers integrated imaging and physical-health features to estimate ASD likelihood. Model performance (accuracy, sensitivity, specificity, precision, and 95% confidence intervals) was averaged across repetitions.

Our methodology constructs a machine learning classification pipeline designed to assign imaging and physical health markers to one of two categories: ASD or non-ASD. This pipeline is structured into three principal modules: (i) model fitting; (ii) propensity-scores-based cohort matching and model evaluation; and (iii) marker importance assessment. A diagram outlining this pipeline is shown in Fig. 2. The subsequent section offers a detailed explanation of each module in the pipeline.

#### 2.6.1 Cross-Validation and Metrics

We implemented stratified K-fold cross-validation (CV), distributing individual subjects into k mutually exclusive folds while maintaining an even distribution of ASD and non-ASD participants across these folds. In each iteration of the CV, data from K-1 folds were utilized to train a model that distinguishes between ASD and non-ASD as binary classes. Predictions were then made for the subjects in the remaining fold. This process ensured that each subject diagnosed with ASD was at least once in the fold excluded from training, receiving a prediction. The classification effectiveness was measured by the area under the receiver operating characteristic curve (AUC-ROC) and the precision-recall curve (AUC-PR), along with predictive accuracy, sensitivity, and specificity (decision threshold set at 0.5). To account for variability in sampling, this cross-validation was performed multiple times with different random partitions of the folds. Non-parametric confidence intervals for the AUC-ROC and AUC-PR were calculated using the 2.5th and 97.5th percentiles across these multiple repetitions.

#### 2.6.2 Propensity-Score Matching

To mitigate demographic confounding and address the pronounced class imbalance between ASD and non-ASD participants, we employed a propensity-score–based matching strategy within the CV framework. Propensity scores were obtained directly from the ABCD dataset and are derived from demographic variables including age, gender, and race/ethnicity, enabling demographic alignment while remaining independent of the imaging and physical-health features used for model training.

In each CV iteration, the dataset was first partitioned into training and validation folds. Propensity-score matching was then performed independently within each fold to construct demographically balanced ASD and non-ASD subsets. Specifically, matched subsets were generated using a k-nearest neighbors algorithm in propensity-score space, whereby for each ASD participant, one non-ASD individual was randomly selected from among its nearest neighbors. As matching was conducted separately within the training and validation folds, no individual could appear simultaneously in both matched sets, thereby eliminating the possibility of information leakage.

**Fig. 3.**
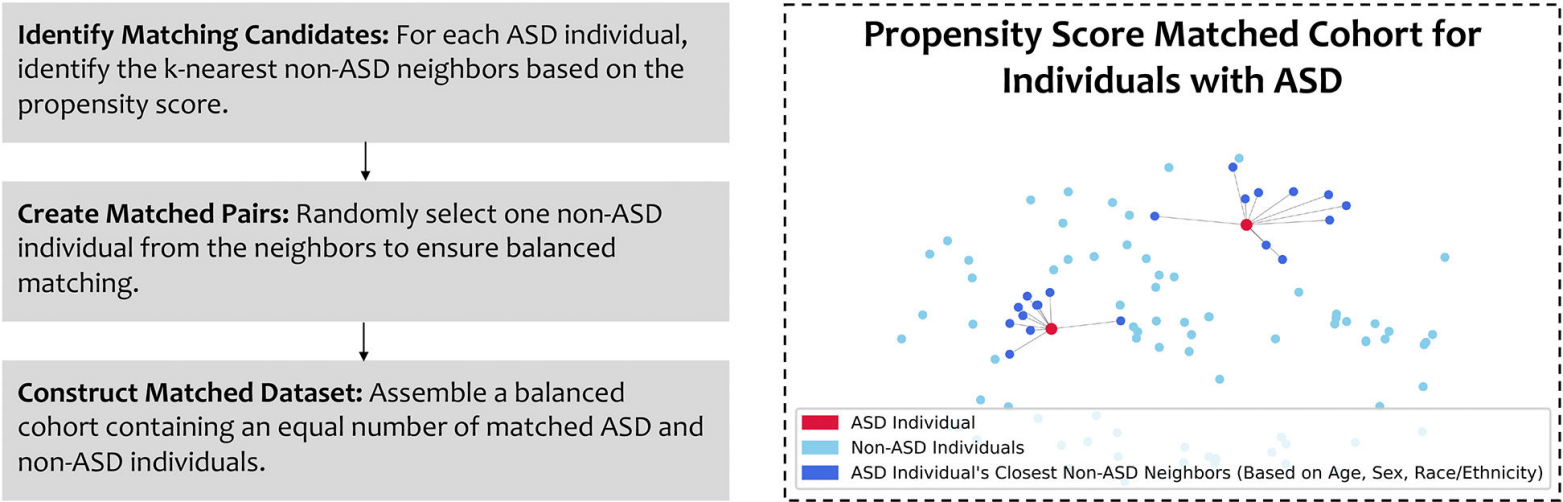
Propensity-score matching procedure. Stepwise schematic illustrating the construction of demographically balanced ASD and non-ASD subsets using propensity scores provided by the ABCD study and based on age, sex, and race/ethnicity. In each cross-validation iteration, participants were first divided into training and validation folds. Propensity-score matching was then performed independently within each fold using a k-nearest neighbors approach, whereby for each ASD participant, one non-ASD individual was randomly selected from among its nearest neighbors in propensity-score space. This procedure was repeated across cross-validation folds and repetitions to generate multiple randomized matched datasets used for ensemble training and balanced model evaluation.

This stochastic matching procedure was repeated independently across CV folds and repetitions, yielding multiple distinct matched datasets rather than a single fixed matched cohort. In the training folds, multiple randomized matched subsets were used to train individual classifiers that were subsequently aggregated into an ensemble, while matched validation subsets were used exclusively for performance evaluation. By averaging model performance across many independently matched realizations, this approach reduces sensitivity to individual control selection and yields statistically robust performance estimates (Fig. 3).

#### 2.6.3 Preprocessing and Feature Selection

In each CV iteration, continuous value markers were standardized by subtracting the mean and dividing by the standard deviation, calculated from the training folds data. We applied Pearson correlation to identify linear correlations between pairs of markers in the training folds. For highly correlated markers – those with a Pearson correlation coefficient surpassing a predefined threshold P, one was randomly selected for exclusion from the analysis during that specific k-fold CV iteration. Feature selection was determined using training data only within each CV iteration, and the resulting feature set was then applied unchanged to the corresponding held-out fold to avoid information leakage. Dimensionality reduction and decomposition methods such as principal component analysis (PCA) and independent component analysis (ICA) were not applied in order to preserve marker-level interpretability and facilitate feature-level importance analyses.

#### 2.6.4 Classifier and Ensembling Strategy

An ensemble of logistic regression models was employed to establish a mapping function linking the imaging and physical health markers to the predefined ASD binary outcome. Each logistic regression model was trained with L2 (ridge) regularization to mitigate overfitting in the high-dimensional feature setting. In each CV iteration, N randomized matched cohorts were generated from the training folds. Each of these cohorts served as the training subset for individual members of the ensemble, employing a bagging technique to enhance model robustness.

#### 2.6.5 Marker Importance via Shapley Values

Shapley value analysis was used to determine the individual importance of markers for specific predictions and their overall relevance across the validation folds. This assessment was based on the arithmetic mean of the absolute Shapley values.

#### 2.6.6 Implementation Details

All statistical and machine learning analyses were conducted in Python (version ≥3.10, Python Software Foundation, Wilmington, DE, USA, https://python.org). Classification models were implemented using scikit-learn (version 1.8.0; https://scikit-learn.org), with an ensemble of L2-regularizedlogisticregressionmodelsservingastheprimary classifier. Feature standardization, correlation-based feature pruning, propensity-score matching via k-nearest neighbors, and stratified cross-validation procedures were implemented using scikit-learn utilities. Model performance metrics, including AUC-ROC, AUC-PR, accuracy, sensitivity, and specificity, were computed using scikit-learn metrics (version 1.8.0; https://scikit-learn.org/stable/modules/model_evaluation.html).

Shapley value analyses for marker importance were performed using the SHAP library (version 0.50.0; https://github.com/shap/shap). Data handling and numerical computations were conducted using NumPy (version 2.3.1; https://numpy.org) and pandas (version 2.3.3; https://pandas.pydata.org). Visualization was performed using matplotlib (version 3.10.0; https://matplotlib.org) and seaborn (version 0.13.2; https://seaborn.pydata.org). All analyses used fixed random seeds within each repetition to ensure reproducibility.

The code is hardware-agnostic; computations were performed on a workstation equipped with an NVIDIA GeForce RTX 2080 Ti GPU (NVIDIA Corporation, Santa Clara, CA, USA), although GPU acceleration was not required for model training.

## 3. Results

### 3.1 Study Population Characteristics

Of the 201 ASD cases reported in the full ABCD cohort, 118 met prespecified baseline eligibility criteria after exclusions for incomplete imaging or physical health markers, imaging quality control, and history of traumatic brain injury or loss of consciousness (Fig. 1). The final analytic sample comprised 8503 participants from the ABCD cohort, including 118 children with parent-reported ASD (1.4%) and 8385 non-ASD controls (98.6%). Participants were recruited across 22 study sites, with a mean (±SD) of 381.1 ± 167.9 non-ASD and 5.6 ± 2.4 ASD participants per site.

The non-ASD group showed an approximately balanced sex distribution (50.7% female, 49.3% male), whereas the ASD group was predominantly male (82.2% male, 17.8% female). Racial and ethnic composition in the two groups included a majority identifying as White (54.4% non-ASD; 54.2% ASD), followed by Hispanic (20.4% non-ASD; 16.9% ASD), Black (13.2% non-ASD; 11.9% ASD), Asian (1.8% non-ASD; 0.8% ASD), and Other racial/ethnic categories (10.3% non-ASD; 16.1% ASD).

The mean (±SD) age at interview was 9.93 ± 0.63 years for non-ASD participants and 10.00 ± 0.61 years for ASD participants. Interviews were conducted between September 2016 and October 2018. Demographic variables including age, sex, and race/ethnicity were subsequently accounted for using propensity-score matching during model training and evaluation.

### 3.2 *ASD vs. Non-ASD Classification Performance* ROC/PR Across Modalities

**Fig. 4.**
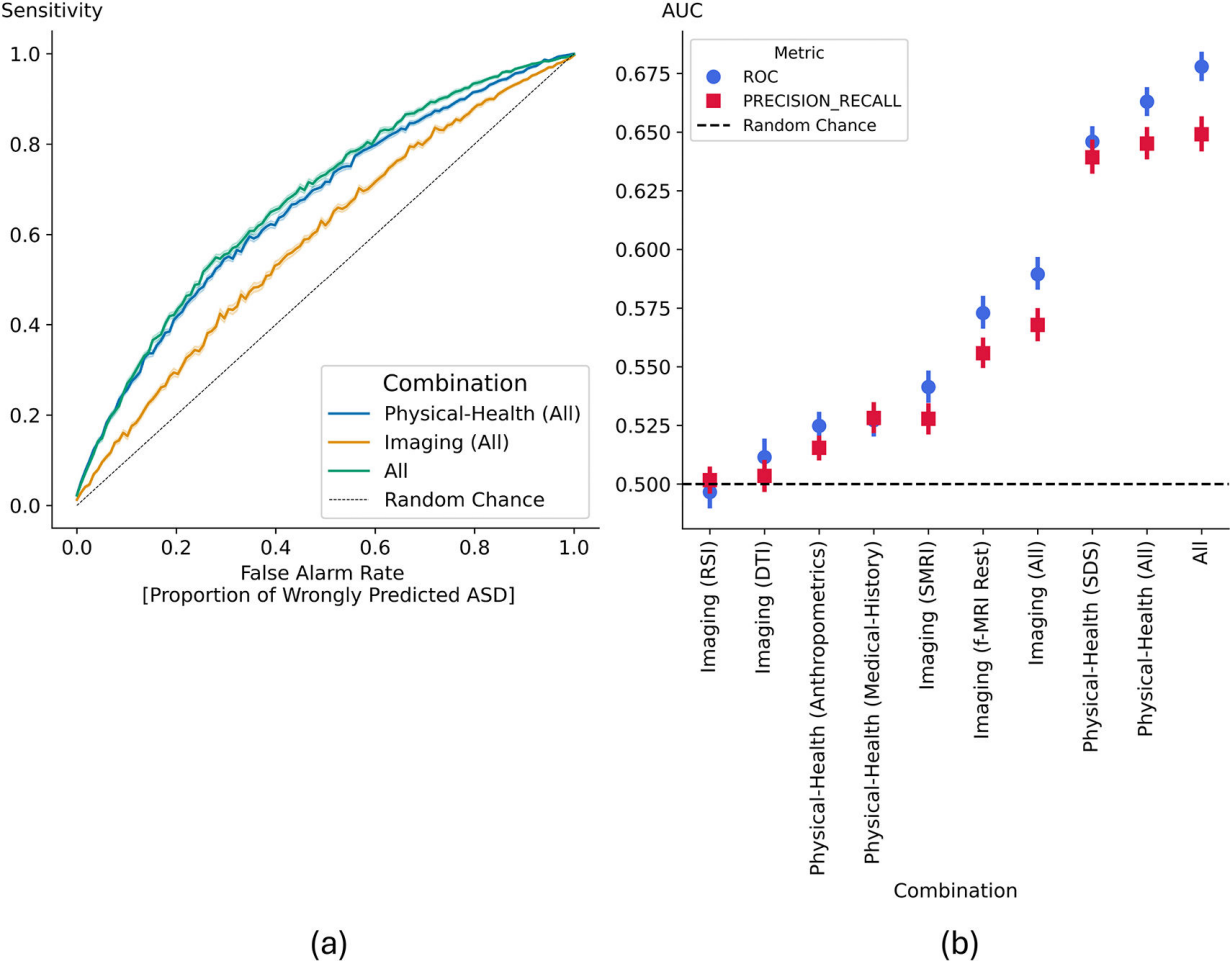
Model performance across modalities. (a) ROC curves for imaging-only, physical-health–only, and multimodal (combined) models. (b) AUC for ROC and precision-recall (PR) curves across all imaging and physical-health tasks. Error bars denote standard deviation (SD) across cross-validation repetitions.

The machine learning pipeline was applied to each marker category. 300 repetitions of 5-fold cross validation and 10-nearest neighbors’ propensity-score matching were applied. Marker pairs with absolute Pearson correlation higher than *p* = 0.90 were considered highly correlated for feature selection purposes. N = 10 logistic regression models constructed an ensemble. The classification results detailing the different marker combinations are presented in Table 1 and Fig. 4b.

The receiver operating characteristic curves for the categories including all imaging and physical-health markers as well as their combination are presented in Fig. 4a. The multimodal combination including all imaging and physical-health markers achieved the highest classification performance: median AUC-ROC (95% CI) and AUC-PR (95% CI) of 0.68 (0.62, 0.73) and 0.66 (0.60, 0.73) respectively. The unimodality imaging-only and physical-health only categories achieved AUC-ROC (95% CI) of 0.59 (0.53, 0.64) and 0.66 (0.62, 0.71) and AUC-PR (95% CI) 0.58 (0.53, 0.64) and 0.66 (0.60, 0.71) respectively. The worst performing imaging-based classification was obtained for the RSI biomarkers with median AUC-ROC (95% CI) and AUC-PR (95% CI) of 0.50 (0.43, 0.56) and 0.51 (0.47, 0.58) respectively. The worst performing physical-health-based classification was obtained for the Anthropometrics markers with median AUC-ROC (95% CI) and AUC-PR (95% CI) of 0.52 (0.47, 0.57) and 0.53 (0.49, 0.57). Both were not statistically different from chance level.

### 3.3 Feature Importance

#### 3.3.1 Top Imaging Biomarkers (SHAP)

Table 2 lists the ten most influential imaging biomarkers from the imaging-only model and their corresponding ranks in both the imaging-only and combined-modality (imaging + physical-health) models. It also indicates the direction of each feature’s contribution, where (+) values correspond to a higher likelihood of ASD and (−) values correspond to a lower likelihood. The cortical thickness (mm) of Automated Anatomical Parcellation (APARC) ROI rh-superiortemporal (structural biomarker) and the average correlation between cingulo-opercular network and default network (functional biomarker) had the most impact on the final classification in both the imaging-only category and for the combined imaging and physical-health category. Both, indicated an increased likelihood of ASD classification. In total, 8 out of 10 the most important imaging biomarkers in the imaging-only model were also among the 10 most important imaging biomarkers in the combined-modality model, half of them had a positive coefficient indicating a contribution to the likelihood of ASD and half of them with a negative coefficient indicating a lower likelihood of ASD.

#### 3.3.2 Cross-Modality Contributions

**Fig. 5.**
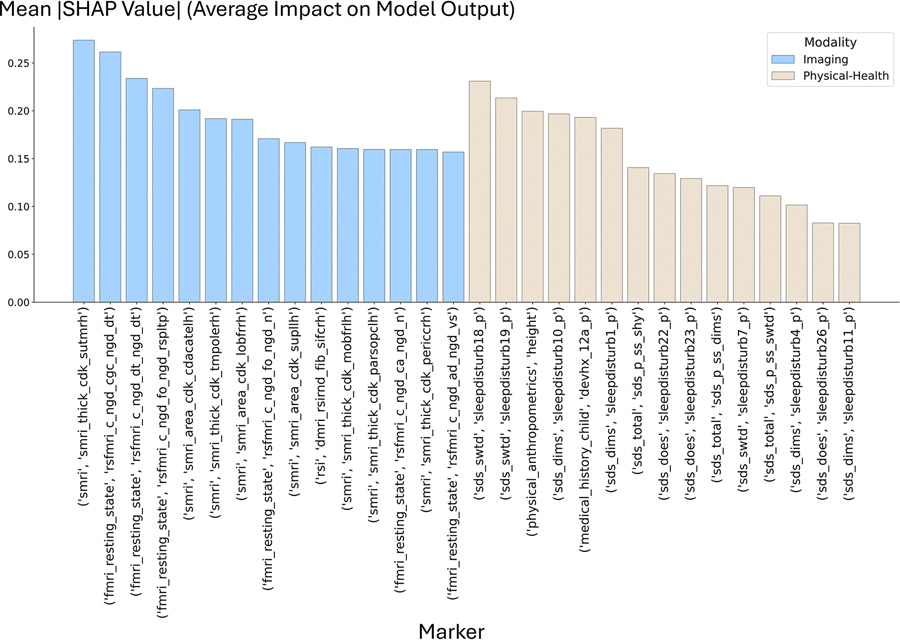
Top 15 most influential biomarkers from imaging and physical-health categories contributing to ASD classification. Bar plots show the mean absolute SHAP values representing the average impact of each biomarker on model output in the combined model. The left panel displays imaging biomarkers, and the right panel displays physical-health biomarkers. Higher SHAP values indicate greater influence on ASD classification.

Top 15-most important markers in each modality – imaging, and physical-health, in the ‘combined-modality’ model and their mean absolute Shapley values appear in Fig. 5. Among the 10-most important features in the ‘combined-modality’ model, 5 were imaging biomarkers (M = 3 f-MRI and M = 2 s-MRI), and 5 physical-health markers (M = 3 SDSC, M = 1 anthropometric indicating the relative height in the matched cohorts, and M=1 medical history marker indicating whether the child was born prematurely).

## 4. Discussion

In this retrospective study, we used the ABCD dataset to examine the contributions of neuroimaging and physical-health markers, both individually and in combination, to ASD classification using a machine learning (ML) approach. Our findings show that integrating multimodal data improves classification performance compared to individual modalities, highlighting the complementary role of imaging and clinical features. Sleep function emerged as the most informative physical-health marker, while functional connectivity from resting-state f-MRI was the strongest neuroimaging predictor. These results emphasize the value of multimodal approaches and large-scale datasets in advancing ASD classification and improving our understanding of its neurobiological and physiological characteristics.

### 4.1 Comparison With Prior Work

Our study demonstrates the utility of the ABCD dataset in evaluating multimodal approaches for ASD classification. The combined modality approach, integrating both neuroimaging and physical-health markers, achieved an AUC-ROC of 0.68 (95% CI: 0.62–0.73). While this performance is slightly lower than results from specialized ASD datasets, it underscores the challenge of ASD classification in a large, heterogeneous population.

Importantly, this level of performance should be interpreted in the context of ABCD’s population-based design. Unlike ASD-enriched clinical cohorts, ABCD reflects a community sample with substantial heterogeneity in symptom profiles, comorbidities, and diagnostic certainty, which is expected to reduce separability between ASD and non-ASD participants. In addition, ASD is relatively underrepresented even in a large cohort, which limits statistical power. Viewed through this lens, the observed AUCROC values likely represent a conservative estimate of multimodal signal in a real-world developmental population.

Previous multimodal studies have reported higher performance within curated ASD cohorts. For instance, Zhang *et al*. [27] combined f-MRI and s-MRI data from 114 Autism Brain Imaging Data Exchange (ABIDE) subjects, achieving an AUC-ROC of 0.73. Similarly, Song *et al*. [28] employed a dual-transformer graph convolutional network to integrate functional and structural MRI data, reporting an accuracy of 79.47% on ABIDE-I and 76.55% on ABIDE-II. Wang *et al*. [29] leveraged functional MRI and phenotypic data from the ABIDE dataset, achieving an AUC-ROC of 0.78 in a multi-site, multimodal analysis of 449 individuals. While these studies demonstrate promising results, they are often limited to well-characterized ASD cohorts, which may not fully capture the heterogeneity seen in real-world clinical settings. In contrast, the ABCD dataset provides several key advantages, including population-representative sampling, comprehensive multimodal assessments, and a longitudinal design. These factors enable the development and validation of ASD classification models that may better generalize to diverse clinical populations and developmental trajectories.

Recent meta-analyses provide further context for this interpretation. Although higher pooled AUC-ROC values have been reported [30], these analyses primarily summarize ASD–control classification studies conducted in curated clinical cohorts and note substantial heterogeneity, small-study effects, and reduced performance in larger, multi-site datasets. Together, these findings underscore the challenges of translating optimized classification performance to population-representative developmental samples such as ABCD.

In light of these considerations, the machine learning pipeline used in the present study was intentionally designed to prioritize interpretability, robustness, and suitability for population-based data rather than maximizing classification performance. Unlike many prior ASD classification studies that employ complex deep learning architectures trained on curated clinical cohorts, we adopted a regularized and transparent modeling framework combined with systematic modality ablation and feature-level interpretation. This approach enables direct comparison of the relative contributions of neuroimaging and physical-health markers under substantial phenotypic heterogeneity and diagnostic uncertainty.

Accordingly, beyond absolute performance metrics, the primary contribution of this work lies in its population-based, multimodal framework for characterizing ASD-related biological signal. Using a large, community-based sample, we show that physical-health markers, particularly sleep-related measures, contribute meaningfully to ASD classification and provide complementary information when integrated with neuroimaging. The modest classification performance observed here is therefore itself informative, highlighting the limitations of MRI-centric approaches in population settings and motivating integrative, interpretable strategies for studying ASD in real-world developmental cohorts.

### 4.2 Neurobiological Interpretation

Our findings identify several structural differences in ASD (Table 2). The Shapley analysis revealed positive contributions from cortical thickness in the right superior temporal gyrus and right temporal pole. These findings are consistent with multiple prior studies: Jou *et al*. [31] found significantly increased right superior temporal gyral volumes in autism, Shen *et al*. [32] identified thicker cortical thickness in the right superior temporal gyrus, Patriquin *et al*. [33] confirmed increased cortical thickness in ASD, and Pereira *et al*. [34] specifically found increased cortical thickness in the right temporal pole. Additionally, we found negative Shapley values for cortical surface area in both the left caudal anterior cingulate cortex and left superior parietal cortex, consistent with Ecker *et al*. [35] findings of decreased surface area in the anterior cingulate. These structural alterations suggest potential neuroanatomical differences in ASD that warrant further investigation.

Our findings also highlight several functional connectivity markers associated with ASD. The Shapley analysis revealed increased functional connectivity between the cingulo-opercular network (CON) and the default mode network (DMN), consistent with de Lacy *et al*. [36], who reported hyperconnectivity between these networks in individuals with ASD. Additionally, we observed a reduction in intranetwork connectivity within the DMN, aligning with Assaf *et al*. [37], who reported reduced connectivity within DMN sub-networks in ASD. Furthermore, our analysis indicated reduced fronto-parietal network (FPN) connectivity, which is supported by Yerys *et al*. [38], who reported weaker FPN functional connectivity in children with ASD compared to typically developing controls. However, we could not find specific literature supporting a direct functional connection between the cingulo-opercular network and the sensorimotor hand network or between the frontoparietal network and the retrosplenial temporal network in ASD. These connectivity patterns may represent novel findings that warrant further investigation to determine their relevance to ASD pathophysiology.

### 4.3 Physical-Health Findings (Sleep, Prematurity, Growth)

Our findings indicate that preterm birth and sleep disturbances are associated with ASD, consistent with prior research. The positive correlation with premature birth aligns with Crump *et al.* [39], who reported higher ASD prevalence in preterm individuals, ranging from 6.1% in extremely preterm (22–27 weeks) to 1.4% in full-term (39–41 weeks) births. Additionally, frequent night waking and bruxism were positively associated with ASD, in line with studies reporting a high prevalence of sleep disturbances in ASD populations. Romeo *et al.* [40] found that 46% of autistic children had abnormal scores on at least one SDSC factor, with overall sleep disorder prevalence ranging from 45% to 86%. Hodge *et al*. [41] similarly reported that children with ASD were more likely to have sleep problems than typically developing children. We also observed a positive correlation with height, though prior research on this relationship is inconclusive. Green *et al*. [42] found indications of overgrowth in ASD populations, but most studies focused on early childhood, with limited data on later development. Conversely, talking in sleep was negatively associated with ASD. While no direct literature addresses this relationship, sleep talking could be indicative of a well-developed sleep architecture and language processing system, which may be less typical in ASD individuals. Given that ASD often involves delayed or atypical language development, sleep talking might reflect a more typical verbal processing pattern. Further research is needed to explore this potential link.

### 4.4 Limitations

First, ASD diagnosis in the dataset is based on parental report rather than clinical evaluation, which may introduce variability in case identification. Although this approach is less precise than gold-standard diagnostic instruments, prior work within the ABCD framework has shown that parent-reported ASD diagnosis captures measurable neurodevelopmental and clinical signal at the population level, including prediction of downstream psychopathology consistent with known ASD-related risk trajectories [15]. Importantly, non-differential diagnostic misclassification would be expected to attenuate effect sizes and classification performance rather than inflate them. Accordingly, the modest classification performance observed in this study likely represents a conservative estimate of the true multimodal signal present in a large, community-based cohort.

Second, although the ABCD dataset offers a large and demographically diverse cohort, ASD remains relatively underrepresented, potentially affecting model generalizability. To partially mitigate class imbalance and confounding by demographic variables, we applied propensity-score cohort matching during model training and evaluation. However, residual biases may still exist. More broadly, model performance was assessed using internal cross-validation within ABCD, and no external dataset was available for independent validation. While ABCD is a large, multi-site study with harmonized imaging protocols and cross-validation folds include participants drawn from multiple imaging centers, indicating the presence of shared, non-site-specific signal, this form of internal validation does not establish generalizability to independent populations, acquisition protocols, or diagnostic frameworks. Accordingly, the reported results should be interpreted as cohort-specific, and future studies incorporating external datasets will be necessary to assess external validity.

Third, our ML approach focuses on classification performance rather than causal inference, limiting conclusions about underlying mechanisms. While we examined a broad set of neuroimaging and physical-health markers, other potentially informative modalities, such as genetic and environmental factors, were not included. Incorporation of such high-dimensional modalities in ABCD is constrained by substantial sparsity across subjects, as many measures are not uniformly available, and their inclusion would reduce the effective ASD sample size and limit statistical power in a population-based cohort. In addition, despite the comprehensive imaging protocol in ABCD, scan quality and site-related variability may introduce additional noise.

Finally, the practical implications of the observed classification performance warrant careful consideration. Although incorporation of MRI features resulted in a modest improvement in classification performance, the absolute AUC remains limited and should not be interpreted as evidence of immediate clinical utility. The analyses presented here should therefore be viewed as exploratory rather than confirmatory, with observed differences intended to be descriptive and hypothesis-generating. Future studies incorporating clinically validated ASD diagnoses, larger ASD samples, and additional biological and behavioral data may further refine predictive models and enhance their interpretability.

## 5. Conclusions

This study is the first to leverage the ABCD dataset—a large, population-based cohort of adolescents—for a comprehensive multimodal analysis of ASD. By integrating structural and functional neuroimaging with physical-health markers, our work provides a novel perspective on ASD classification and its underlying biomarkers at the population level, rather than focusing on high-risk cohorts. Our analysis highlights the value of combining modalities: while unimodal contributions, such as cortical thickness in the right superior temporal gyrus and functional connectivity between the cingulo-opercular and default mode networks, reaffirm known ASD biomarkers, the integration of multiple modalities significantly enhances classification performance. Through systematic ablation studies, we demonstrate the complementary roles of neuroimaging and physical-health features, with multimodal approaches outperforming unimodal ones. Additionally, we identify less-studied imaging biomarkers and connectivity patterns that may open new directions for understanding ASD’s neurobiological heterogeneity. These findings underscore the potential of large-scale, multimodal datasets like ABCD to refine our understanding of ASD and provide a foundation for more targeted diagnostic and therapeutic strategies. Future research can build on this work by incorporating additional biological and environmental data, as well as clinically validated diagnoses, to further enhance the precision and interpretability of predictive models.

## Supplementary Material

Supplementary Material

Supplementary material associated with this article can be found, in the online version, at https://??.

## Figures and Tables

**Table 1.** Classification results for ASD across data modalities and tasks.

Modality	Task	Markers (mean selected)	AUC-ROC median (95% CI)	AUC-PR median (95% CI)	Accuracy median (95% CI)	Sensitivity median (95% CI)	Specificity median (95% CI)

Imaging	RSI	37 (20.9)	0.50 (0.43, 0.56)	0.51 (0.47, 0.58)	0.50 (0.45, 0.55)	0.47 (0.39, 0.55)	0.53 (0.45, 0.62)
Imaging	DTI	148 (49.1)	0.51 (0.44, 0.58)	0.51 (0.47, 0.57)	0.51 (0.44, 0.58)	0.50 (0.42, 0.57)	0.53 (0.42, 0.63)
Physical-Health	Anthropometrics	4 (2.0)	0.52 (0.47, 0.57)	0.53 (0.49, 0.57)	0.51 (0.47, 0.56)	0.47 (0.42, 0.51)	0.56 (0.48, 0.65)
Physical-Health	Medical-History	3 (2.3)	0.53 (0.47, 0.59)	0.54 (0.48, 0.60)	0.53 (0.48, 0.58)	0.40 (0.32, 0.46)	0.67 (0.58, 0.76)
Imaging	s-MRI	136 (135.3)	0.54 (0.49, 0.60)	0.54 (0.49, 0.59)	0.53 (0.49, 0.58)	0.51 (0.45, 0.58)	0.54 (0.46, 0.62)
Imaging	f-MRI Rest	91 (91.0)	0.57 (0.51, 0.64)	0.56 (0.53, 0.63)	0.55 (0.49, 0.61)	0.54 (0.47, 0.61)	0.55 (0.47, 0.64)
Imaging	All	412 (276.0)	0.59 (0.53, 0.64)	0.58 (0.53, 0.64)	0.57 (0.51,0.63)	0.56 (0.50, 0.63)	0.58 (0.50, 0.67)
Physical-Health	SDSC	33 (30.0)	0.65 (0.58, 0.70)	0.66 (0.58, 0.71)	0.62 (0.57, 0.67)	0.56 (0.50, 0.62)	0.68 (0.57, 0.75)
Physical-Health	All	40 (33.3)	0.66 (0.62, 0.71)	0.66 (0.60, 0.71)	0.62 (0.57, 0.67)	0.58 (0.52, 0.64)	0.66 (0.58, 0.73)
All	All	452 (312.4)	0.68 (0.62, 0.73)	0.66 (0.60, 0.73)	0.63 (0.58, 0.68)	0.62 (0.54, 0.68)	0.64 (0.56, 0.73)

Values represent median (95% confidence interval) performance across repeated cross-validation and propensity-score–matched cohorts. Modalities are ordered by AUC-ROC.

Abbreviations: RSI, restriction spectrum imaging; DTI, diffusion tensor imaging; s-MRI, structural magnetic resonance imaging; f-MRI, functional magnetic resonance imaging; SDSC, Sleep Disturbance Scale for Children; AUC-ROC, area under the receiver operating characteristic curve; AUC-PR, area under the precision-recall curve; CI, confidence interval.

**Table 2.** Top 10 imaging biomarkers contributing to ASD classification.

Imaging Biomarker			Biomarker Importance (mean|SHAP|)		

Task	ABCD Variable Name	ABCD Description	Regression Coefficient Sign	Imaging Only Rank	Combined-Modality Rank
*s*-MRI	thick_cdk_sutmrh	Cortical thickness in mm of APARC ROI rh-superiortemporal	+	1 [most important]	1
*f*-MRI	c_ngd_cgc_ngd_dt	Average correlation between cingulo-opercular network and default network	+	2	2
*f*-MRI	c_ngd_cgc_ngd_smh	Average correlation between cingulo-opercular network and sensorimotor hand network	+	3	18
*s*-MRI	thick_cdk_tmpolerh	Cortical thickness in mm of APARC ROI rh-temporalpole	+	4	6
*s*-MRI	area_cdk_cdacatelh	Cortical area in mm^2^ of APARC ROI lh-caudalanteriorcingulate	−	5	5
*f*-MRI	c_ngd_dt_ngd_dt	Average correlation between default network and default network	−	6	3
*f*-MRI	c_ngd_ca_ngd_n	Average correlation between cingulo-parietal network and none network	−	7	13
*f*-MRI	c_ngd_fo_ngd_rspltp	Average correlation between fronto-parietal network and retrosplenial temporal network	+	8	4
*s*-MRI	area_cdk_supllh	Cortical area in mm^2^ of APARC ROI lh-superiorparietal	−	9	9
*f*-MRI	C_ngd_fo_ngd_n	Average correlation between fronto-parietal network and none network	−	10	8

Listed biomarkers were identified by SHAP analysis as the most influential predictors in the imaging-only model. The table also reports their corresponding ranks in the combined-modality model that integrated imaging and physical-health markers. Positive and negative regression-coefficient signs indicate whether higher biomarker values were associated with an increased or decreased likelihood of ASD classification, respectively. Both the “ABCD Variable Name” and “ABCD Description” columns follow the definitions provided in the ABCD Study data dictionary.

Abbreviations: APARC, Automated Anatomical Parcellation; ROI, region of interest.

## Data Availability

Data used in the preparation of this article were obtained from the Adolescent Brain Cognitive Development (ABCD) Study (https://abcdstudy.org), held in the NIMH Data Archive (NDA). This is a multisite, longitudinal study designed to recruit more than 10,000 children age 9–10 and follow them over 10 years into early adulthood. The ABCD data repository grows and changes over time. The ABCD data used in this report came from 10.15154/z563-zd24. DOIs can be found at https://nda.nih.gov/abcd/abcd-annual-releases.
