# Supplementary material for: Integrating Multimodal Neuroimaging and Physical-Health Markers for Autism Spectrum Disorder in the ABCD Study
Source: J Integr Neurosci. Author manuscript; Available in PMC 2026 Mar 26. (PMC13019123; doi:10.31083/JIN48212)
Supplement: Supplementary Material [file NIHMS2153118-supplement-Supplementary_Material.pdf]

Supplementary Material

This file provides detailed information on the biomarkers used in the study "Integrating Multimodal Neuroimaging and Physical-Health Markers for Autism Spectrum Disorder in the ABCD Study."

Supplementary Table S1: Physical-Health Markers Descriptions

- Description: Each row corresponds to one biomarker included in the analysis.
- Columns:
  - Modality: physical-health domain (e.g., anthropometrics, SDSC, developmental history)
  - Variable name: the ABCD variable identifier used in the dataset
  - Meaning: a short explanation of the biomarker or measure
- Source: Extracted from the ABCD Data Repository (Release 5.1, DOI: 10.15154/z563-zd24).
- Usage: Used for feature selection and model interpretation in the multimodal classification analyses.

**Modality**	**Sub-Task**	**Variable Name**	**Meaning**
Anthropometrics	physical_anthropometrics	anthroheightcalc	Standing Height Average (inches): If three measurements were obtained, the two closest measurements will be averaged.
	physical_anthropometrics	anthroweightcalc	Average Measured Weight (lbs):If three measurements were obtained, the two closest measurements will be averaged.
	physical_anthropometrics	anthro_waist_cm	Measured circumference (in inches):
Sleep Disorders Specialty (SDS)	sds_dims	sleepdisturb1_p	How many hours of sleep does your child get on most nights?
	sds_dims	sleepdisturb2_p	How long after going to bed does your child usually fall asleep?
	sds_dims	sleepdisturb3_p	The child goes to bed reluctantly.
	sds_dims	sleepdisturb4_p	The child has difficulty getting to sleep at night.
	sds_dims	sleepdisturb5_p	The child feels anxious or afraid when falling asleep.
	sds_dims	sleepdisturb10_p	The child wakes up more than twice per night.
	sds_dims	sleepdisturb11_p	After waking up in the night, the child has difficulty to fall asleep again.
	sds_swtd	sleepdisturb6_p	The child startles or jerks parts of the body while falling asleep.
	sds_swtd	sleepdisturb7_p	The child shows repetitive actions such as rocking or head banging while falling asleep.
	sds_swtd	sleepdisturb8_p	The child experiences vivid dream-like scenes while falling asleep.
	sds_swtd	sleepdisturb12_p	The child has frequent twitching or jerking of legs while asleep or often changes position during the night or kicks the covers off the bed.
	sds_swtd	sleepdisturb18_p	You have observed the child talking in their sleep.
	sds_swtd	sleepdisturb19_p	The child grinds their teeth during sleep.
	sds_shy	sleepdisturb9_p	The child sweats excessively while falling asleep.
	sds_shy	sleepdisturb16_p	The child sweats excessively during the night. El niño / la niña suda en exceso durante la noche.
	sds_sbd	sleepdisturb13_p	The child has difficulty in breathing during the night. El niño / la niña tiene dificultad respirando durante la noche.
	sds_sbd	sleepdisturb14_p	The child gasps for breaths or is unable to breathe during sleep. El niño / la niña toma bocanadas de aire para respirar o no puede respirar mientras duerme.
	sds_sbd	sleepdisturb15_p	The child snores. El niño / la niña ronca.
	sds_da	sleepdisturb17_p	You have observed the child sleepwalking.
	sds_da	sleepdisturb20_p	The child wakes from sleep screaming or confused so you cannot seem to get through to they, but has no memory of these events the next morning.
	sds_da	sleepdisturb21_p	The child has nightmares which they don't remember the next day.
	sds_does	sleepdisturb22_p	The child is unusuallly difficult to wake up in the morning.
	sds_does	sleepdisturb23_p	The child awakes in the morning feeling tired.
	sds_does	sleepdisturb24_p	The child feels unable to move when waking up in the morning.
	sds_does	sleepdisturb25_p	The child experiences daytime sleepiness.

	sds_does	sleepdisturb26_p	The child falls asleep suddenly in inappropriate situations.
	sds_total	sds_p_ss_dims	Disorders of Initiating and Maintaining Sleep (DIMS) SUM: sleepdisturb1_p + sleepdisturb2_p + sleepdisturb3_p + sleepdisturb4_p + sleepdisturb5_p + sleepdisturb10_p + sleepdisturb11_p; Validation: All items must be answered
	sds_total	sds_p_ss_sbd	Sleep Breathing disorders (SBD): SUM sleepdisturb13_p + sleepdisturb14_p + sleepdisturb15_p; Validation: All items must be answered
	sds_total	sds_p_ss_da	Disorder of Arousal (DA) SUM: sleepdisturb17_p + sleepdisturb20_p + sleepdisturb21_p; Validation: All items must be answered
	sds_total	sds_p_ss_swtd	Sleep-Wake transition Disorders (SWTD) SUM: sleepdisturb6_p + sleepdisturb7_p + sleepdisturb8_p + sleepdisturb12_p + sleepdisturb18_p + sleepdisturb19_p; Validation: All items must be answered
	sds_total	sds_p_ss_does	Disorders of Excessive Somnolence (DOES) SUM: sleepdisturb22_p + sleepdisturb23_p + sleepdisturb24_p + sleepdisturb25_p + sleepdisturb26_p; Validation: All items must be answered
	sds_total	sds_p_ss_shy	Sleep Hyperhydrosis (SHY) SUM: sleepdisturb9_p + sleepdisturb16_p; Validation: All items must be answered
	sds_total	sds_p_ss_total	Total Score (Sum of 6 Factors): sds_p_ss_dims + sds_p_ss_sbd + sds_p_ss_da + sds_p_ss_swtd + sds_p_ss_does + sds_p_ss_shy; Validation: All items must be answered
Medical-History	medical_history_mom	devhx_3_p	How old were you/biological mother when the child was born?
	medical_history_child	devhx_12a_p	Was the child born prematurely?
	medical_history_child	devhx_12_p	About how many weeks premature was the child when they were born?

Supplementary Table S2: Neuroimaging Biomarkers Descriptions

- Description: Each row corresponds to one biomarker included in the analysis.
- Columns:
  - Task: imaging modality (e.g., sMRI, dMRI, fMRI)
  - Variable name: the ABCD variable identifier used in the dataset
  - Meaning: a short explanation of the biomarker or measure
- Source: Extracted from the ABCD Data Repository (Release 5.1, DOI: 10.15154/z563-zd24).
- Usage: Used for feature selection and model interpretation in the multimodal classification analyses.

Task	Variable Name	Meaning
DTI	dmdtifp1_10	Average fractional anisotropy within DTI atlas tract left anterior thalamic radiations
	dmdtifp1_9	Average fractional anisotropy within DTI atlas tract right anterior thalamic radiations
	dmdtifp1_19	Average fractional anisotropy within DTI atlas tract corpus callosum
	dmdtifp1_4	Average fractional anisotropy within DTI atlas tract left cingulate cingulum
	dmdtifp1_3	Average fractional anisotropy within DTI atlas tract right cingulate cingulum
	dmdtifp1_6	Average fractional anisotropy within DTI atlas tract left parahippocampal cingulum
	dmdtifp1_5	Average fractional anisotropy within DTI atlas tract right parahippocampal cingulum
	dmdtifp1_8	Average fractional anisotropy within DTI atlas tract left corticospinal/pyramidal
	dmdtifp1_7	Average fractional anisotropy within DTI atlas tract right corticospinal/pyramidal
	dmdtifp1_17	Average fractional anisotropy within DTI atlas tract foreceps major
	dmdtifp1_18	Average fractional anisotropy within DTI atlas tract foreceps minor
	dmdtifp1_29	Average fractional anisotropy within DTI atlas tract left superior corticostriate-frontal cortex only
	dmdtifp1_28	Average fractional anisotropy within DTI atlas tract right superior corticostriate-frontal cortex only
	dmdtifp1_37	Average fractional anisotropy within DTI atlas tract left fornix, excluding fimbria
	dmdtifp1_36	Average fractional anisotropy within DTI atlas tract right fornix, excluding fimbria
	dmdtifp1_2	Average fractional anisotropy within DTI atlas tract left fornix
	dmdtifp1_1	Average fractional anisotropy within DTI atlas tract right fornix
	dmdtifp1_16	Average fractional anisotropy within DTI atlas tract left inferior-fronto-occipital fasiculus
	dmdtifp1_15	Average fractional anisotropy within DTI atlas tract right inferior-fronto-occipital fasiculus
	dmdtifp1_35	Average fractional anisotropy within DTI atlas tract left inferior frontal superior frontal cortex
	dmdtifp1_34	Average fractional anisotropy within DTI atlas tract right inferior frontal superior frontal cortex
	dmdtifp1_14	Average fractional anisotropy within DTI atlas tract left inferior longitudinal fasiculus
	dmdtifp1_13	Average fractional anisotropy within DTI atlas tract right inferior longitudinal fasiculus
	dmdtifp1_31	Average fractional anisotropy within DTI atlas tract left superior corticostriate-parietal cortex only
	dmdtifp1_30	Average fractional anisotropy within DTI atlas tract right superior corticostriate-parietal cortex only
	dmdtifp1_25	Average fractional anisotropy within DTI atlas tract left parietal superior longitudinal fasiculus
	dmdtifp1_24	Average fractional anisotropy within DTI atlas tract right parietal superior longitudinal fasiculus
	dmdtifp1_27	Average fractional anisotropy within DTI atlas tract left superior corticostriate
	dmdtifp1_26	Average fractional anisotropy within DTI atlas tract right superior corticostriate
	dmdtifp1_33	Average fractional anisotropy within DTI atlas tract left striatal inferior frontal cortex
	dmdtifp1_32	Average fractional anisotropy within DTI atlas tract right striatal inferior frontal cortex
	dmdtifp1_21	Average fractional anisotropy within DTI atlas tract left superior longitudinal fasiculus
	dmdtifp1_20	Average fractional anisotropy within DTI atlas tract right superior longitudinal fasiculus
	dmdtifp1_23	Average fractional anisotropy within DTI atlas tract left temporal superior longitudinal fasiculus
	dmdtifp1_22	Average fractional anisotropy within DTI atlas tract right temporal superior longitudinal fasiculus
	dmdtifp1_12	Average fractional anisotropy within DTI atlas tract left uncinate
	dmdtifp1_11	Average fractional anisotropy within DTI atlas tract right uncinate
	dmdtifp1_94	Average longitudinal diffusion coefficient within DTI atlas tract left anterior thalamic radiations
	dmdtifp1_93	Average longitudinal diffusion coefficient within DTI atlas tract right anterior thalamic radiations
	dmdtifp1_103	Average longitudinal diffusion coefficient within DTI atlas tract corpus callosum

	dmdtifp1_88	Average longitudinal diffusion coefficient within DTI atlas tract left cingulate cingulum
	dmdtifp1_87	Average longitudinal diffusion coefficient within DTI atlas tract right cingulate cingulum
	dmdtifp1_90	Average longitudinal diffusion coefficient within DTI atlas tract left parahippocampal cingulum
	dmdtifp1_89	Average longitudinal diffusion coefficient within DTI atlas tract right parahippocampal cingulum
	dmdtifp1_92	Average longitudinal diffusion coefficient within DTI atlas tract left corticospinal/pyramidal
	dmdtifp1_91	Average longitudinal diffusion coefficient within DTI atlas tract right corticospinal/pyramidal
	dmdtifp1_101	Average longitudinal diffusion coefficient within DTI atlas tract foreceps major
	dmdtifp1_102	Average longitudinal diffusion coefficient within DTI atlas tract foreceps minor
	dmdtifp1_113	Average longitudinal diffusion coefficient within DTI atlas tract left superior corticostriate-frontal cortex only
	dmdtifp1_112	Average longitudinal diffusion coefficient within DTI atlas tract right superior corticostriate-frontal cortex only
	dmdtifp1_121	Average longitudinal diffusion coefficient within DTI atlas tract left fornix, excluding fimbria
	dmdtifp1_120	Average longitudinal diffusion coefficient within DTI atlas tract right fornix, excluding fimbria
	dmdtifp1_86	Average longitudinal diffusion coefficient within DTI atlas tract left fornix
	dmdtifp1_85	Average longitudinal diffusion coefficient within DTI atlas tract right fornix
	dmdtifp1_100	Average longitudinal diffusion coefficient within DTI atlas tract left inferior-fronto-occipital fasiculus
	dmdtifp1_99	Average longitudinal diffusion coefficient within DTI atlas tract right inferior-fronto-occipital fasiculus
	dmdtifp1_119	Average longitudinal diffusion coefficient within DTI atlas tract left inferior frontal superior frontal cortex
	dmdtifp1_118	Average longitudinal diffusion coefficient within DTI atlas tract right inferior frontal superior frontal cortex
	dmdtifp1_98	Average longitudinal diffusion coefficient within DTI atlas tract left inferior longitudinal fasiculus
	dmdtifp1_97	Average longitudinal diffusion coefficient within DTI atlas tract right inferior longitudinal fasiculus
	dmdtifp1_115	Average longitudinal diffusion coefficient within DTI atlas tract left superior corticostriate-parietal cortex only
	dmdtifp1_114	Average longitudinal diffusion coefficient within DTI atlas tract right superior corticostriate-parietal cortex only
	dmdtifp1_109	Average longitudinal diffusion coefficient within DTI atlas tract left parietal superior longitudinal fasiculus
	dmdtifp1_108	Average longitudinal diffusion coefficient within DTI atlas tract right parietal superior longitudinal fasiculus
	dmdtifp1_111	Average longitudinal diffusion coefficient within DTI atlas tract left superior corticostriate
	dmdtifp1_110	Average longitudinal diffusion coefficient within DTI atlas tract right superior corticostriate
	dmdtifp1_117	Average longitudinal diffusion coefficient within DTI atlas tract left striatal inferior frontal cortex
	dmdtifp1_116	Average longitudinal diffusion coefficient within DTI atlas tract right striatal inferior frontal cortex
	dmdtifp1_105	Average longitudinal diffusion coefficient within DTI atlas tract left superior longitudinal fasiculus
	dmdtifp1_104	Average longitudinal diffusion coefficient within DTI atlas tract right superior longitudinal fasiculus
	dmdtifp1_107	Average longitudinal diffusion coefficient within DTI atlas tract left temporal superior longitudinal fasiculus
	dmdtifp1_106	Average longitudinal diffusion coefficient within DTI atlas tract right temporal superior longitudinal fasiculus
	dmdtifp1_96	Average longitudinal diffusion coefficient within DTI atlas tract left uncinate
	dmdtifp1_95	Average longitudinal diffusion coefficient within DTI atlas tract right uncinate
	dmdtifp1_52	Mean diffusivity within DTI atlas tract left anterior thalamic radiations

	dmdtifp1_51	Mean diffusivity within DTI atlas tract right anterior thalamic radiations
	dmdtifp1_61	Mean diffusivity within DTI atlas tract corpus callosum
	dmdtifp1_46	Mean diffusivity within DTI atlas tract left cingulate cingulum
	dmdtifp1_45	Mean diffusivity within DTI atlas tract right cingulate cingulum
	dmdtifp1_48	Mean diffusivity within DTI atlas tract left parahippocampal cingulum
	dmdtifp1_47	Mean diffusivity within DTI atlas tract right parahippocampal cingulum
	dmdtifp1_50	Mean diffusivity within DTI atlas tract left corticospinal/pyramidal
	dmdtifp1_49	Mean diffusivity within DTI atlas tract right corticospinal/pyramidal
	dmdtifp1_59	Mean diffusivity within DTI atlas tract foreceps major
	dmdtifp1_60	Mean diffusivity within DTI atlas tract foreceps minor
	dmdtifp1_71	Mean diffusivity within DTI atlas tract left superior corticostriate-frontal cortex only
	dmdtifp1_70	Mean diffusivity within DTI atlas tract right superior corticostriate-frontal cortex only
	dmdtifp1_79	Mean diffusivity within DTI atlas tract left fornix, excluding fimbria
	dmdtifp1_78	Mean diffusivity within DTI atlas tract right fornix, excluding fimbria
	dmdtifp1_44	Mean diffusivity within DTI atlas tract left fornix
	dmdtifp1_43	Mean diffusivity within DTI atlas tract right fornix
	dmdtifp1_58	Mean diffusivity within DTI atlas tract left inferior-fronto-occipital fasiculus
	dmdtifp1_57	Mean diffusivity within DTI atlas tract right inferior-fronto-occipital fasiculus
	dmdtifp1_77	Mean diffusivity within DTI atlas tract left inferior frontal superior frontal cortex
	dmdtifp1_76	Mean diffusivity within DTI atlas tract right inferior frontal superior frontal cortex
	dmdtifp1_56	Mean diffusivity within DTI atlas tract left inferior longitudinal fasiculus
	dmdtifp1_55	Mean diffusivity within DTI atlas tract right inferior longitudinal fasiculus
	dmdtifp1_73	Mean diffusivity within DTI atlas tract left superior corticostriate-parietal cortex only
	dmdtifp1_72	Mean diffusivity within DTI atlas tract right superior corticostriate-parietal cortex only
	dmdtifp1_67	Mean diffusivity within DTI atlas tract left parietal superior longitudinal fasiculus
	dmdtifp1_66	Mean diffusivity within DTI atlas tract right parietal superior longitudinal fasiculus
	dmdtifp1_69	Mean diffusivity within DTI atlas tract left superior corticostriate
	dmdtifp1_68	Mean diffusivity within DTI atlas tract right superior corticostriate
	dmdtifp1_75	Mean diffusivity within DTI atlas tract left striatal inferior frontal cortex
	dmdtifp1_74	Mean diffusivity within DTI atlas tract right striatal inferior frontal cortex
	dmdtifp1_63	Mean diffusivity within DTI atlas tract left superior longitudinal fasiculus
	dmdtifp1_62	Mean diffusivity within DTI atlas tract right superior longitudinal fasiculus
	dmdtifp1_65	Mean diffusivity within DTI atlas tract left temporal superior longitudinal fasiculus
	dmdtifp1_64	Mean diffusivity within DTI atlas tract right temporal superior longitudinal fasiculus
	dmdtifp1_54	Mean diffusivity within DTI atlas tract left uncinate
	dmdtifp1_53	Mean diffusivity within DTI atlas tract right uncinate
	dmdtifp1_136	Average transverse diffusion coefficient within DTI atlas tract left anterior thalamic radiations
	dmdtifp1_135	Average transverse diffusion coefficient within DTI atlas tract right anterior thalamic radiations
	dmdtifp1_145	Average transverse diffusion coefficient within DTI atlas tract corpus callosum
	dmdtifp1_130	Average transverse diffusion coefficient within DTI atlas tract left cingulate cingulum
	dmdtifp1_129	Average transverse diffusion coefficient within DTI atlas tract right cingulate cingulum
	dmdtifp1_132	Average transverse diffusion coefficient within DTI atlas tract left parahippocampal cingulum

	dmdtifp1_131	Average transverse diffusion coefficient within DTI atlas tract right parahippocampal cingulum
	dmdtifp1_134	Average transverse diffusion coefficient within DTI atlas tract left corticospinal/pyramidal
	dmdtifp1_133	Average transverse diffusion coefficient within DTI atlas tract right corticospinal/pyramidal
	dmdtifp1_143	Average transverse diffusion coefficient within DTI atlas tract foreceps major
	dmdtifp1_144	Average transverse diffusion coefficient within DTI atlas tract foreceps minor
	dmdtifp1_155	Average transverse diffusion coefficient within DTI atlas tract left superior corticostriate-frontal cortex only
	dmdtifp1_154	Average transverse diffusion coefficient within DTI atlas tract right superior corticostriate-frontal cortex only
	dmdtifp1_163	Average transverse diffusion coefficient within DTI atlas tract left fornix, excluding fimbria
	dmdtifp1_162	Average transverse diffusion coefficient within DTI atlas tract right fornix, excluding fimbria
	dmdtifp1_128	Average transverse diffusion coefficient within DTI atlas tract left fornix
	dmdtifp1_127	Average transverse diffusion coefficient within DTI atlas tract right fornix
	dmdtifp1_142	Average transverse diffusion coefficient within DTI atlas tract left inferior-fronto-occipital fasiculus
	dmdtifp1_141	Average transverse diffusion coefficient within DTI atlas tract right inferior-fronto-occipital fasiculus
	dmdtifp1_161	Average transverse diffusion coefficient within DTI atlas tract left inferior frontal superior frontal cortex
	dmdtifp1_160	Average transverse diffusion coefficient within DTI atlas tract right inferior frontal superior frontal cortex
	dmdtifp1_140	Average transverse diffusion coefficient within DTI atlas tract left inferior longitudinal fasiculus
	dmdtifp1_139	Average transverse diffusion coefficient within DTI atlas tract right inferior longitudinal fasiculus
	dmdtifp1_157	Average transverse diffusion coefficient within DTI atlas tract left superior corticostriate-parietal cortex only
	dmdtifp1_156	Average transverse diffusion coefficient within DTI atlas tract right superior corticostriate-parietal cortex only
	dmdtifp1_151	Average transverse diffusion coefficient within DTI atlas tract left parietal superior longitudinal fasiculus
	dmdtifp1_150	Average transverse diffusion coefficient within DTI atlas tract right parietal superior longitudinal fasiculus
	dmdtifp1_153	Average transverse diffusion coefficient within DTI atlas tract left superior corticostriate
	dmdtifp1_152	Average transverse diffusion coefficient within DTI atlas tract right superior corticostriate
	dmdtifp1_159	Average transverse diffusion coefficient within DTI atlas tract left striatal inferior frontal cortex
	dmdtifp1_158	Average transverse diffusion coefficient within DTI atlas tract right striatal inferior frontal cortex
	dmdtifp1_147	Average transverse diffusion coefficient within DTI atlas tract left superior longitudinal fasiculus
	dmdtifp1_146	Average transverse diffusion coefficient within DTI atlas tract right superior longitudinal fasiculus
	dmdtifp1_149	Average transverse diffusion coefficient within DTI atlas tract left temporal superior longitudinal fasiculus
	dmdtifp1_148	Average transverse diffusion coefficient within DTI atlas tract right temporal superior longitudinal fasiculus
	dmdtifp1_138	Average transverse diffusion coefficient within DTI atlas tract left uncinate
	dmdtifp1_137	Average transverse diffusion coefficient within DTI atlas tract right uncinate
rsi	dmri_rsirnd_fib_fxrh	restricted normalized directional diffusion AtlasTrack fiber segmentation right fornix
	dmri_rsirnd_fib_fxlh	restricted normalized directional diffusion AtlasTrack fiber segmentation left fornix
	dmri_rsirnd_fib_cgcrh	restricted normalized directional diffusion AtlasTrack fiber segmentation right cingulate cingulum
	dmri_rsirnd_fib_cgclh	restricted normalized directional diffusion AtlasTrack fiber segmentation left cingulate cingulum

	dmri_rsirnd_fib_cghrh	restricted normalized directional diffusion AtlasTrack fiber segmentation right parahpcm cingulum
	dmri_rsirnd_fib_cghlh	restricted normalized directional diffusion AtlasTrack fiber segmentation left parahpcm cingulum
	dmri_rsirnd_fib_cstrh	restricted normalized directional diffusion AtlasTrack fiber segmentation right corticospinal/pyramidal
	dmri_rsirnd_fib_cstlh	restricted normalized directional diffusion AtlasTrack fiber segmentation left corticospinal/pyramidal
	dmri_rsirnd_fib_atrrh	restricted normalized directional diffusion AtlasTrack fiber segmentation right anterior thalamic radiations
	dmri_rsirnd_fib_atrlh	restricted normalized directional diffusion AtlasTrack fiber segmentation left right anterior thalamic radiations
	dmri_rsirnd_fib_uncrh	restricted normalized directional diffusion AtlasTrack fiber segmentation right uncinate fasciculus
	dmri_rsirnd_fib_unclh	restricted normalized directional diffusion AtlasTrack fiber segmentation left uncinate
	dmri_rsirnd_fib_ilfrh	restricted normalized directional diffusion AtlasTrack fiber segmentation right inferior longitudinal fasciculus
	dmri_rsirnd_fib_ilflh	restricted normalized directional diffusion AtlasTrack fiber segmentation left inferior longitudinal fasciculus
	dmri_rsirnd_fib_iforh	restricted normalized directional diffusion AtlasTrack fiber segmentation right inferior fronto-occipital fasciculus
	dmri_rsirnd_fib_ifolh	restricted normalized directional diffusion AtlasTrack fiber segmentation left inferior fronto-occipital fasciculus
	dmri_rsirnd_fib_fmaj	restricted normalized directional diffusion AtlasTrack fiber segmentation forceps major
	dmri_rsirnd_fib_fmin	restricted normalized directional diffusion AtlasTrack fiber segmentation forceps minor
	dmri_rsirnd_fib_cc	restricted normalized directional diffusion AtlasTrack fiber segmentation corpus callosum
	dmri_rsirnd_fib_slfrh	restricted normalized directional diffusion AtlasTrack fiber segmentation right superior longitudinal fasciculus
	dmri_rsirnd_fib_slflh	restricted normalized directional diffusion AtlasTrack fiber segmentation left superior longitudinal fasciculus
	dmri_rsirnd_fib_tslfrh	restricted normalized directional diffusion AtlasTrack fiber segmentation right temporal superior longitudinal fasiculus
	dmri_rsirnd_fib_tslflh	restricted normalized directional diffusion AtlasTrack fiber segmentation left temporal superior longitudinal fasiculus
	dmri_rsirnd_fib_pslfrh	restricted normalized directional diffusion AtlasTrack fiber segmentation right parietal superior longitudinal fasiculus
	dmri_rsirnd_fib_pslflh	restricted normalized directional diffusion AtlasTrack fiber segmentation left parietal superior longitudinal fasiculus
	dmri_rsirnd_fib_scsrh	restricted normalized directional diffusion AtlasTrack fiber segmentation right superior corticostriate
	dmri_rsirnd_fib_scslh	restricted normalized directional diffusion AtlasTrack fiber segmentation left superior corticostriate
	dmri_rsirnd_fib_fscsrh	restricted normalized directional diffusion AtlasTrack fiber segmentation right superior corticostriate-frontal cortex only
	dmri_rsirnd_fib_fscslh	restricted normalized directional diffusion AtlasTrack fiber segmentation left superior corticostriate-frontal cortex only
	dmri_rsirnd_fib_pscsrh	restricted normalized directional diffusion AtlasTrack fiber segmentation right superior corticostriate-parietal cortex only
	dmri_rsirnd_fib_pscslh	restricted normalized directional diffusion AtlasTrack fiber segmentation left superior corticostriate-parietal cortex only
	dmri_rsirnd_fib_sifcrh	restricted normalized directional diffusion AtlasTrack fiber segmentation right striatal inferior frontal cortex
	dmri_rsirnd_fib_sifclh	restricted normalized directional diffusion AtlasTrack fiber segmentation left striatal inferior frontal cortex
	dmri_rsirnd_fib_ifsfcrh	restricted normalized directional diffusion AtlasTrack fiber segmentation right inferior frontal superior frontal cortex
	dmri_rsirnd_fib_ifsfclh	restricted normalized directional diffusion AtlasTrack fiber segmentation left inferior frontal superior frontal cortex
	dmri_rsirnd_fib_fxcutrh	restricted normalized directional diffusion AtlasTrack fiber segmentation right fornix, excluding fimbria
	dmri_rsirnd_fib_fxcutlh	restricted normalized directional diffusion AtlasTrack fiber segmentation left fornix, excluding fimbria
fmri_resting_state	rsfmri_c_ngd_ad_ngd_ad	Average correlation between auditory network and auditory network

	rsfmri_c_ngd_ad_ngd_cgc	Average correlation between auditory network and cingulo-opercular network
	rsfmri_c_ngd_ad_ngd_ca	Average correlation between auditory network and cingulo-parietal network
	rsfmri_c_ngd_ad_ngd_dt	Average correlation between auditory network and default network
	rsfmri_c_ngd_ad_ngd_dla	Average correlation between auditory network and dorsal attention network
	rsfmri_c_ngd_ad_ngd_fo	Average correlation between auditory network and fronto-parietal network
	rsfmri_c_ngd_ad_ngd_n	Average correlation between auditory network and none network
	rsfmri_c_ngd_ad_ngd_rspltp	Average correlation between auditory network and retrosplenial temporal network
	rsfmri_c_ngd_ad_ngd_sa	Average correlation between auditory network and salience network
	rsfmri_c_ngd_ad_ngd_smh	Average correlation between auditory network and sensorimotor hand network
	rsfmri_c_ngd_ad_ngd_smm	Average correlation between auditory network and sensorimotor mouth network
	rsfmri_c_ngd_ad_ngd_vta	Average correlation between auditory network and ventral attention network
	rsfmri_c_ngd_ad_ngd_vs	Average correlation between auditory network and visual network
	rsfmri_c_ngd_cgc_ngd_cgc	Average correlation between cingulo-opercular network and cingulo-opercular network
	rsfmri_c_ngd_cgc_ngd_ca	Average correlation between cingulo-opercular network and cingulo-parietal network
	rsfmri_c_ngd_cgc_ngd_dt	Average correlation between cingulo-opercular network and default network
	rsfmri_c_ngd_cgc_ngd_dla	Average correlation between cingulo-opercular network and dorsal attention network
	rsfmri_c_ngd_cgc_ngd_fo	Average correlation between cingulo-opercular network and fronto-parietal network
	rsfmri_c_ngd_cgc_ngd_n	Average correlation between cingulo-opercular network and none network
	rsfmri_c_ngd_cgc_ngd_rspltp	Average correlation between cingulo-opercular network and retrosplenial temporal network
	rsfmri_c_ngd_cgc_ngd_sa	Average correlation between cingulo-opercular network and salience network
	rsfmri_c_ngd_cgc_ngd_smh	Average correlation between cingulo-opercular network and sensorimotor hand network
	rsfmri_c_ngd_cgc_ngd_smm	Average correlation between cingulo-opercular network and sensorimotor mouth network
	rsfmri_c_ngd_cgc_ngd_vta	Average correlation between cingulo-opercular network and ventral attention network
	rsfmri_c_ngd_cgc_ngd_vs	Average correlation between cingulo-opercular network and visual network
	rsfmri_c_ngd_ca_ngd_ca	Average correlation between cingulo-parietal network and cingulo-parietal network
	rsfmri_c_ngd_ca_ngd_dt	Average correlation between cingulo-parietal network and default network
	rsfmri_c_ngd_ca_ngd_dla	Average correlation between cingulo-parietal network and dorsal attention network
	rsfmri_c_ngd_ca_ngd_fo	Average correlation between cingulo-parietal network and fronto-parietal network
	rsfmri_c_ngd_ca_ngd_n	Average correlation between cingulo-parietal network and none network
	rsfmri_c_ngd_ca_ngd_rspltp	Average correlation between cingulo-parietal network and retrosplenial temporal network
	rsfmri_c_ngd_ca_ngd_sa	Average correlation between cingulo-parietal network and salience network
	rsfmri_c_ngd_ca_ngd_smh	Average correlation between cingulo-parietal network and sensorimotor hand network
	rsfmri_c_ngd_ca_ngd_smm	Average correlation between cingulo-parietal network and sensorimotor mouth network
	rsfmri_c_ngd_ca_ngd_vta	Average correlation between cingulo-parietal network and ventral attention network
	rsfmri_c_ngd_ca_ngd_vs	Average correlation between cingulo-parietal network and visual network
	rsfmri_c_ngd_dt_ngd_dt	Average correlation between default network and default network
	rsfmri_c_ngd_dt_ngd_dla	Average correlation between default network and dorsal attention network
	rsfmri_c_ngd_dt_ngd_fo	Average correlation between default network and fronto-parietal network
	rsfmri_c_ngd_dt_ngd_n	Average correlation between default network and none network
	rsfmri_c_ngd_dt_ngd_rspltp	Average correlation between default network and retrosplenial temporal network
	rsfmri_c_ngd_dt_ngd_sa	Average correlation between default network and salience network
	rsfmri_c_ngd_dt_ngd_smh	Average correlation between default network and sensorimotor hand network

	rsfmri_c_ngd_dt_ngd_smm	Average correlation between default network and sensorimotor mouth network
	rsfmri_c_ngd_dt_ngd_vta	Average correlation between default network and ventral attention network
	rsfmri_c_ngd_dt_ngd_vs	Average correlation between default network and visual network
	rsfmri_c_ngd_dla_ngd_dla	Average correlation between dorsal attention network and dorsal attention network
	rsfmri_c_ngd_dla_ngd_fo	Average correlation between dorsal attention network and fronto-parietal network
	rsfmri_c_ngd_dla_ngd_n	Average correlation between dorsal attention network and none network
	rsfmri_c_ngd_dla_ngd_rspltp	Average correlation between dorsal attention network and retrosplenial temporal network
	rsfmri_c_ngd_dla_ngd_sa	Average correlation between dorsal attention network and salience network
	rsfmri_c_ngd_dla_ngd_smh	Average correlation between dorsal attention network and sensorimotor hand network
	rsfmri_c_ngd_dla_ngd_smm	Average correlation between dorsal attention network and sensorimotor mouth network
	rsfmri_c_ngd_dla_ngd_vta	Average correlation between dorsal attention network and ventral attention network
	rsfmri_c_ngd_dla_ngd_vs	Average correlation between dorsal attention network and visual network
	rsfmri_c_ngd_fo_ngd_fo	Average correlation between fronto-parietal network and fronto-parietal network
	rsfmri_c_ngd_fo_ngd_n	Average correlation between fronto-parietal network and none network
	rsfmri_c_ngd_fo_ngd_rspltp	Average correlation between fronto-parietal network and retrosplenial temporal network
	rsfmri_c_ngd_fo_ngd_sa	Average correlation between fronto-parietal network and salience network
	rsfmri_c_ngd_fo_ngd_smh	Average correlation between fronto-parietal network and sensorimotor hand network
	rsfmri_c_ngd_fo_ngd_smm	Average correlation between fronto-parietal network and sensorimotor mouth network
	rsfmri_c_ngd_fo_ngd_vta	Average correlation between fronto-parietal network and ventral attention network
	rsfmri_c_ngd_fo_ngd_vs	Average correlation between fronto-parietal network and visual network
	rsfmri_c_ngd_n_ngd_n	Average correlation between none network and none network
	rsfmri_c_ngd_n_ngd_rspltp	Average correlation between none network and retrosplenial temporal network
	rsfmri_c_ngd_n_ngd_sa	Average correlation between none network and salience network
	rsfmri_c_ngd_n_ngd_smh	Average correlation between none network and sensorimotor hand network
	rsfmri_c_ngd_n_ngd_smm	Average correlation between none network and sensorimotor mouth network
	rsfmri_c_ngd_n_ngd_vta	Average correlation between none network and ventral attention network
	rsfmri_c_ngd_n_ngd_vs	Average correlation between none network and visual network
	rsfmri_c_ngd_rspltp_ngd_rspltp	Average correlation between retrosplenial temporal network and retrosplenial temporal network
	rsfmri_c_ngd_rspltp_ngd_sa	Average correlation between retrosplenial temporal network and salience network
	rsfmri_c_ngd_rspltp_ngd_smh	Average correlation between retrosplenial temporal network and sensorimotor hand network
	rsfmri_c_ngd_rspltp_ngd_smm	Average correlation between retrosplenial temporal network and sensorimotor mouth network
	rsfmri_c_ngd_rspltp_ngd_vta	Average correlation between retrosplenial temporal network and ventral attention network
	rsfmri_c_ngd_rspltp_ngd_vs	Average correlation between retrosplenial temporal network and visual network
	rsfmri_c_ngd_sa_ngd_sa	Average correlation between salience network and salience network
	rsfmri_c_ngd_sa_ngd_vta	Average correlation between salience network and ventral attention network
	rsfmri_c_ngd_sa_ngd_vs	Average correlation between salience network and visual network
	rsfmri_c_ngd_smh_ngd_sa	Average correlation between sensorimotor hand network and salience network
	rsfmri_c_ngd_smh_ngd_smh	Average correlation between sensorimotor hand network and sensorimotor hand network
	rsfmri_c_ngd_smh_ngd_smm	Average correlation between sensorimotor hand network and sensorimotor mouth network
	rsfmri_c_ngd_smh_ngd_vta	Average correlation between sensorimotor hand network and ventral attention network
	rsfmri_c_ngd_smh_ngd_vs	Average correlation between sensorimotor hand network and visual network

	rsfmri_c_ngd_smm_ngd_sa	Average correlation between sensorimotor mouth network and salience network
	rsfmri_c_ngd_smm_ngd_smm	Average correlation between sensorimotor mouth network and sensorimotor mouth network
	rsfmri_c_ngd_smm_ngd_vta	Average correlation between sensorimotor mouth network and ventral attention network
	rsfmri_c_ngd_smm_ngd_vs	Average correlation between sensorimotor mouth network and visual network
	rsfmri_c_ngd_vta_ngd_vta	Average correlation between ventral attention network and ventral attention network
	rsfmri_c_ngd_vta_ngd_vs	Average correlation between ventral attention network and visual network
	rsfmri_c_ngd_vs_ngd_vs	Average correlation between visual network and visual network
smri	smri_area_cdk_banksstslh	Cortical area in mm^2 of APARC ROI lh-Banks of Superior Temporal Sulcus
	smri_area_cdk_cdacatelh	Cortical area in mm^2 of APARC ROI lh-caudalanteriorcingulate
	smri_area_cdk_cdmdfrlh	Cortical area in mm^2 of APARC ROI lh-caudalmiddlefrontal
	smri_area_cdk_cuneuslh	Cortical area in mm^2 of APARC ROI lh-cuneus
	smri_area_cdk_ehinallh	Cortical area in mm^2 of APARC ROI lh-entorhinal
	smri_area_cdk_fusiformlh	Cortical area in mm^2 of APARC ROI lh-fusiform
	smri_area_cdk_ifpllh	Cortical area in mm^2 of APARC ROI lh-inferiorparietal
	smri_area_cdk_iftmlh	Cortical area in mm^2 of APARC ROI lh-inferiortemporal
	smri_area_cdk_ihcatelh	Cortical area in mm^2 of APARC ROI lh-isthmuscingulate
	smri_area_cdk_locclh	Cortical area in mm^2 of APARC ROI lh-lateraloccipital
	smri_area_cdk_lobfrlh	Cortical area in mm^2 of APARC ROI lh-lateralorbitofrontal
	smri_area_cdk_linguallh	Cortical area in mm^2 of APARC ROI lh-lingual
	smri_area_cdk_mobfrlh	Cortical area in mm^2 of APARC ROI lh-medialorbitofrontal
	smri_area_cdk_mdtmlh	Cortical area in mm^2 of APARC ROI lh-middletemporal
	smri_area_cdk_parahpallh	Cortical area in mm^2 of APARC ROI lh-parahippocampal
	smri_area_cdk_paracnlh	Cortical area in mm^2 of APARC ROI lh-paracentral
	smri_area_cdk_parsopclh	Cortical area in mm^2 of APARC ROI lh-parsopercularis
	smri_area_cdk_parsobislh	Cortical area in mm^2 of APARC ROI lh-parsorbitalis
	smri_area_cdk_parstgrislh	Cortical area in mm^2 of APARC ROI lh-parstriangularis
	smri_area_cdk_pericclh	Cortical area in mm^2 of APARC ROI lh-pericalcarine
	smri_area_cdk_postcnlh	Cortical area in mm^2 of APARC ROI lh-postcentral
	smri_area_cdk_ptcatelh	Cortical area in mm^2 of APARC ROI lh-posteriorcingulate
	smri_area_cdk_precnlh	Cortical area in mm^2 of APARC ROI lh-precentral
	smri_area_cdk_pclh	Cortical area in mm^2 of APARC ROI lh-precuneus
	smri_area_cdk_rracatelh	Cortical area in mm^2 of APARC ROI lh-rostralanteriorcingulate
	smri_area_cdk_rrmdfrlh	Cortical area in mm^2 of APARC ROI lh-rostralmiddlefrontal
	smri_area_cdk_sufrlh	Cortical area in mm^2 of APARC ROI lh-superiorfrontal
	smri_area_cdk_supllh	Cortical area in mm^2 of APARC ROI lh-superiorparietal
	smri_area_cdk_sutmlh	Cortical area in mm^2 of APARC ROI lh-superiortemporal
	smri_area_cdk_smlh	Cortical area in mm^2 of APARC ROI lh-supramarginal
	smri_area_cdk_frpolelh	Cortical area in mm^2 of APARC ROI lh-frontalpole
	smri_area_cdk_tmpolelh	Cortical area in mm^2 of APARC ROI lh-temporalpole
	smri_area_cdk_trvtmlh	Cortical area in mm^2 of APARC ROI lh-transversetemporal
	smri_area_cdk_insulalh	Cortical area in mm^2 of APARC ROI lh-insula
	smri_area_cdk_banksstsrh	Cortical area in mm^2 of APARC ROI rh-Banks of Superior Temporal Sulcus
	smri_area_cdk_cdacaterh	Cortical area in mm^2 of APARC ROI rh-caudalanteriorcingulate

	smri_area_cdk_cdmdfrrh	Cortical area in mm^2 of APARC ROI rh-caudalmiddlefrontal
	smri_area_cdk_cuneusrh	Cortical area in mm^2 of APARC ROI rh-cuneus
	smri_area_cdk_ehinalrh	Cortical area in mm^2 of APARC ROI rh-entorhinal
	smri_area_cdk_fusiformrh	Cortical area in mm^2 of APARC ROI rh-fusiform
	smri_area_cdk_ifplrh	Cortical area in mm^2 of APARC ROI rh-inferiorparietal
	smri_area_cdk_iftmrh	Cortical area in mm^2 of APARC ROI rh-inferiortemporal
	smri_area_cdk_ihcaterh	Cortical area in mm^2 of APARC ROI rh-isthmuscingulate
	smri_area_cdk_loccrh	Cortical area in mm^2 of APARC ROI rh-lateraloccipital
	smri_area_cdk_lobfrrh	Cortical area in mm^2 of APARC ROI rh-lateralorbitofrontal
	smri_area_cdk_lingualrh	Cortical area in mm^2 of APARC ROI rh-lingual
	smri_area_cdk_mobfrrh	Cortical area in mm^2 of APARC ROI rh-medialorbitofrontal
	smri_area_cdk_mdtmrh	Cortical area in mm^2 of APARC ROI rh-middletemporal
	smri_area_cdk_parahpalrh	Cortical area in mm^2 of APARC ROI rh-parahippocampal
	smri_area_cdk_paracnrh	Cortical area in mm^2 of APARC ROI rh-paracentral
	smri_area_cdk_parsopcrh	Cortical area in mm^2 of APARC ROI rh-parsopercularis
	smri_area_cdk_parsobisrh	Cortical area in mm^2 of APARC ROI rh-parsorbitalis
	smri_area_cdk_parstgrisrh	Cortical area in mm^2 of APARC ROI rh-parstriangularis
	smri_area_cdk_periccrh	Cortical area in mm^2 of APARC ROI rh-pericalcarine
	smri_area_cdk_postcnrh	Cortical area in mm^2 of APARC ROI rh-postcentral
	smri_area_cdk_ptcaterh	Cortical area in mm^2 of APARC ROI rh-posteriorcingulate
	smri_area_cdk_precnrh	Cortical area in mm^2 of APARC ROI rh-precentral
	smri_area_cdk_pcrh	Cortical area in mm^2 of APARC ROI rh-precuneus
	smri_area_cdk_rracaterh	Cortical area in mm^2 of APARC ROI rh-rostralanteriorcingulate
	smri_area_cdk_rrmdfrrh	Cortical area in mm^2 of APARC ROI rh-rostralmiddlefrontal
	smri_area_cdk_sufrrh	Cortical area in mm^2 of APARC ROI rh-superiorfrontal
	smri_area_cdk_suplrh	Cortical area in mm^2 of APARC ROI rh-superiorparietal
	smri_area_cdk_sutmrh	Cortical area in mm^2 of APARC ROI rh-superiortemporal
	smri_area_cdk_smrh	Cortical area in mm^2 of APARC ROI rh-supramarginal
	smri_area_cdk_frpolerh	Cortical area in mm^2 of APARC ROI rh-frontalpole
	smri_area_cdk_tmpolerh	Cortical area in mm^2 of APARC ROI rh-temporalpole
	smri_area_cdk_trvtmrh	Cortical area in mm^2 of APARC ROI rh-transversetemporal
	smri_area_cdk_insularh	Cortical area in mm^2 of APARC ROI rh-insula
	smri_thick_cdk_banksstslh	Cortical thickness in mm of APARC ROI lh-Banks of Superior Temporal Sulcus
	smri_thick_cdk_cdacatelh	Cortical thickness in mm of APARC ROI lh-caudalanteriorcingulate
	smri_thick_cdk_cdmdfrlh	Cortical thickness in mm of APARC ROI lh-caudalmiddlefrontal
	smri_thick_cdk_cuneuslh	Cortical thickness in mm of APARC ROI lh-cuneus
	smri_thick_cdk_ehinallh	Cortical thickness in mm of APARC ROI lh-entorhinal
	smri_thick_cdk_fusiformlh	Cortical thickness in mm of APARC ROI lh-fusiform
	smri_thick_cdk_ifpllh	Cortical thickness in mm of APARC ROI lh-inferiorparietal
	smri_thick_cdk_iftmlh	Cortical thickness in mm of APARC ROI lh-inferiortemporal
	smri_thick_cdk_ihcatelh	Cortical thickness in mm of APARC ROI lh-isthmuscingulate
	smri_thick_cdk_locclh	Cortical thickness in mm of APARC ROI lh-lateraloccipital
	smri_thick_cdk_lobfrlh	Cortical thickness in mm of APARC ROI lh-lateralorbitofrontal

	smri_thick_cdk_linguallh	Cortical thickness in mm of APARC ROI lh-lingual
	smri_thick_cdk_mobfrlh	Cortical thickness in mm of APARC ROI lh-medialorbitofrontal
	smri_thick_cdk_mdtmlh	Cortical thickness in mm of APARC ROI lh-middletemporal
	smri_thick_cdk_parahpallh	Cortical thickness in mm of APARC ROI lh-parahippocampal
	smri_thick_cdk_paracnlh	Cortical thickness in mm of APARC ROI lh-paracentral
	smri_thick_cdk_parsopclh	Cortical thickness in mm of APARC ROI lh-parsopercularis
	smri_thick_cdk_parsobislh	Cortical thickness in mm of APARC ROI lh-parsorbitalis
	smri_thick_cdk_parstgrislh	Cortical thickness in mm of APARC ROI lh-parstriangularis
	smri_thick_cdk_pericclh	Cortical thickness in mm of APARC ROI lh-pericalcarine
	smri_thick_cdk_postcnlh	Cortical thickness in mm of APARC ROI lh-postcentral
	smri_thick_cdk_ptcatelh	Cortical thickness in mm of APARC ROI lh-posteriorcingulate
	smri_thick_cdk_precnlh	Cortical thickness in mm of APARC ROI lh-precentral
	smri_thick_cdk_pclh	Cortical thickness in mm of APARC ROI lh-precuneus
	smri_thick_cdk_rracatelh	Cortical thickness in mm of APARC ROI lh-rostralanteriorcingulate
	smri_thick_cdk_rrmdfrlh	Cortical thickness in mm of APARC ROI lh-rostralmiddlefrontal
	smri_thick_cdk_sufrlh	Cortical thickness in mm of APARC ROI lh-superiorfrontal
	smri_thick_cdk_supllh	Cortical thickness in mm of APARC ROI lh-superiorparietal
	smri_thick_cdk_sutmlh	Cortical thickness in mm of APARC ROI lh-superiortemporal
	smri_thick_cdk_smlh	Cortical thickness in mm of APARC ROI lh-supramarginal
	smri_thick_cdk_frpolelh	Cortical thickness in mm of APARC ROI lh-frontalpole
	smri_thick_cdk_tmpolelh	Cortical thickness in mm of APARC ROI lh-temporalpole
	smri_thick_cdk_trvtmlh	Cortical thickness in mm of APARC ROI lh-transversetemporal
	smri_thick_cdk_insulalh	Cortical thickness in mm of APARC ROI lh-insula
	smri_thick_cdk_banksstsrh	Cortical thickness in mm of APARC ROI rh-Banks of Superior Temporal Sulcus
	smri_thick_cdk_cdacaterh	Cortical thickness in mm of APARC ROI rh-caudalanteriorcingulate
	smri_thick_cdk_cdmdfrrh	Cortical thickness in mm of APARC ROI rh-caudalmiddlefrontal
	smri_thick_cdk_cuneusrh	Cortical thickness in mm of APARC ROI rh-cuneus
	smri_thick_cdk_ehinalrh	Cortical thickness in mm of APARC ROI rh-entorhinal
	smri_thick_cdk_fusiformrh	Cortical thickness in mm of APARC ROI rh-fusiform
	smri_thick_cdk_ifplrh	Cortical thickness in mm of APARC ROI rh-inferiorparietal
	smri_thick_cdk_iftmrh	Cortical thickness in mm of APARC ROI rh-inferiortemporal
	smri_thick_cdk_ihcaterh	Cortical thickness in mm of APARC ROI rh-isthmuscingulate
	smri_thick_cdk_loccrh	Cortical thickness in mm of APARC ROI rh-lateraloccipital
	smri_thick_cdk_lobfrrh	Cortical thickness in mm of APARC ROI rh-lateralorbitofrontal
	smri_thick_cdk_lingualrh	Cortical thickness in mm of APARC ROI rh-lingual
	smri_thick_cdk_mobfrrh	Cortical thickness in mm of APARC ROI rh-medialorbitofrontal
	smri_thick_cdk_mdtmrh	Cortical thickness in mm of APARC ROI rh-middletemporal
	smri_thick_cdk_parahpalrh	Cortical thickness in mm of APARC ROI rh-parahippocampal
	smri_thick_cdk_paracnrh	Cortical thickness in mm of APARC ROI rh-paracentral
	smri_thick_cdk_parsopcrh	Cortical thickness in mm of APARC ROI rh-parsopercularis
	smri_thick_cdk_parsobisrh	Cortical thickness in mm of APARC ROI rh-parsorbitalis
	smri_thick_cdk_parstgrisrh	Cortical thickness in mm of APARC ROI rh-parstriangularis
	smri_thick_cdk_periccrh	Cortical thickness in mm of APARC ROI rh-pericalcarine

	smri_thick_cdk_postcnrh	Cortical thickness in mm of APARC ROI rh-postcentral
	smri_thick_cdk_ptcaterh	Cortical thickness in mm of APARC ROI rh-posteriorcingulate
	smri_thick_cdk_precnrh	Cortical thickness in mm of APARC ROI rh-precentral
	smri_thick_cdk_pcrh	Cortical thickness in mm of APARC ROI rh-precuneus
	smri_thick_cdk_rracaterh	Cortical thickness in mm of APARC ROI rh-rostralanteriorcingulate
	smri_thick_cdk_rrmdfrrh	Cortical thickness in mm of APARC ROI rh-rostralmiddlefrontal
	smri_thick_cdk_sufrrh	Cortical thickness in mm of APARC ROI rh-superiorfrontal
	smri_thick_cdk_suplrh	Cortical thickness in mm of APARC ROI rh-superiorparietal
	smri_thick_cdk_sutmrh	Cortical thickness in mm of APARC ROI rh-superiortemporal
	smri_thick_cdk_smrh	Cortical thickness in mm of APARC ROI rh-supramarginal
	smri_thick_cdk_frpolerh	Cortical thickness in mm of APARC ROI rh-frontalpole
	smri_thick_cdk_tmpolerh	Cortical thickness in mm of APARC ROI rh-temporalpole
	smri_thick_cdk_trvtmrh	Cortical thickness in mm of APARC ROI rh-transversetemporal
	smri_thick_cdk_insularh	Cortical thickness in mm of APARC ROI rh-insula
